# Into the Past: A Step Towards a Robust Kimberley Rock Art Chronology

**DOI:** 10.1371/journal.pone.0161726

**Published:** 2016-08-31

**Authors:** June Ross, Kira Westaway, Meg Travers, Michael J. Morwood, John Hayward

**Affiliations:** 1 Department of Archaeology, University of New England, Armidale, New South Wales, Australia; 2 Department of Environmental Sciences, Macquarie University, Sydney, New South Wales, Australia; 3 Centre for Archaeological Science, University of Wollongong, Wollongong, New South Wales, Australia; 4 Department of Parks and Wildlife, Government of Western Australia, Kununurra, Western Australia, Australia; Max-Planck-Institut fur Menschheitsgeschichte, GERMANY

## Abstract

The recent establishment of a minimum age estimate of 39.9 ka for the origin of rock art in Sulawesi has challenged claims that Western Europe was the locus for the production of the world’s earliest art assemblages. Tantalising excavated evidence found across northern Australian suggests that Australia too contains a wealth of ancient art. However, the dating of rock art itself remains the greatest obstacle to be addressed if the significance of Australian assemblages are to be recognised on the world stage. A recent archaeological project in the northwest Kimberley trialled three dating techniques in order to establish chronological markers for the proposed, regional, relative stylistic sequence. Applications using optically-stimulated luminescence (OSL) provided nine minimum age estimates for fossilised mudwasp nests overlying a range of rock art styles, while Accelerator Mass Spectrometry radiocarbon (AMS ^14^C) results provided an additional four. Results confirm that at least one phase of the northwest Kimberley rock art assemblage is Pleistocene in origin. A complete motif located on the ceiling of a rockshelter returned a minimum age estimate of 16 ± 1 ka. Further, our results demonstrate the inherent problems in relying solely on stylistic classifications to order rock art assemblages into temporal sequences. An earlier than expected minimum age estimate for one style and a maximum age estimate for another together illustrate that the Holocene Kimberley rock art sequence is likely to be far more complex than generally accepted with different styles produced contemporaneously well into the last few millennia. It is evident that reliance on techniques that produce minimum age estimates means that many more dating programs will need to be undertaken before the stylistic sequence can be securely dated.

## Introduction

The rock art sequence of the rugged and remote Kimberley region of tropical northwestern Australia is likely to prove one of the longest and most complex anywhere in the world. Rockshelter substrates, where much of the art is located, are ideal for the preservation of paintings being comprised of particularly hard and stable King Leopold Sandstone, a strongly bedded quartzarenite [1]. Although the production of Kimberley rock art spans many thousands of years, unlike the ancient cave art of Europe [2] or the paintings recently dated in Indonesia [3], it remains central to the cultural beliefs of the Indigenous population of the region today. Ethnographic accounts confirm that painting and ‘retouching’ of rock art was still practised well into the twentieth century [4–6]. The relative sequence of Kimberley rock art styles inferred from studies of superimpositions and differential weathering at many hundreds of sites across an area of 423,517 km^2^ shows that there have been changes in artistic conventions, subject matter, context of production and function through time [7–17]. The rock art assemblage thus provides a unique dataset from which to identify changes in social structure, ideology, economic practises, material culture and marine contact across northwestern Australia [11]. While many of these changes are evident in the figurative elements of the art, especially the anthropomorphic figures that dominate the assemblage, they also manifest in the relationships between the art, and the environmental and social contexts in which it was produced. If these changes are to be placed in a temporal framework and set against the excavated evidence of past life-ways, the rock art assemblage needs to be securely dated. Recent excavations in the northwest Kimberley reveal that occupation in the area had been initiated by at least 36 ka indicating that modern humans were in the region by this time and could have potentially been producing art. However, excavated evidence from the southern Kimberley indicates that occupation of the region could be as early as 42yka (O’Connor 1995). The greatest challenge is to align the chronology of the art with the archaeological and contextual evidence.

Prior to our research, there were a limited number of age estimates available on which to anchor the Kimberley rock art sequence and, with one exception, all are of Holocene age [9,18–21]. Pleistocene dates were obtained from two fossilised mudwasp nests using what was at the time, an experimental optical dating technique not previously trialled on rock art–optically-stimulated luminescence (OSL). Quartz grains from a mudwasp nest (KERC4) partially covering a Gwion Period anthropomorphic figure (that overlies a hand stencil and underlies and Painted Hand Period zoomorph) provided a minimum age estimate of 16,400 ± 1,800 years. A slightly older minimum age estimate of 17,500 ± 1,800 years was obtained from the core of the nest (KERC5) adjacent to, but not directly associated with the motif [20]. Subsequently, the dating technique, the structure of the nests and the location of the nests in relation to the Gwion figure have all been challenged [22–25] Although Roberts stands by the technique and the age estimates [24–25], additional dated motifs are required to confirm the antiquity of aspects of the Kimberley assemblage.

Despite the conjecture surrounding the age of the Kimberley assemblage, there is compelling archaeological evidence that suggests that at least some art across northern Australia has a Pleistocene origin. Evidence for the early use of ochre was recovered from excavations in Arnhem Land to the east of the Kimberley where ground ochre ‘crayons’ were recovered from a strata dated to around 53,000 BP using thermo-luminescence (TL) at Nauwalabila I and from strata bracketed by 61,000 ± 13,000 and 45,000 ± 9000 year-old TL age estimates from Malakunanja II (now known as Madjabebe) [26–28].

In the southern Kimberley, a rock slab covered in red haematite was excavated from deposits dated to a minimum of 42,700 BP [29]. In the northeast of Australia in Cape York, a layer of yellow haematite pigment sandwiched between mineral crusts was dated using AMS ^14^C to about 25,000 BP [30]. However, the earliest, generally accepted Australian date for rock art was obtained from a recently excavated slab with an indeterminate black pigment motif recovered at Nawarla Gabarnmang in Arnhem Land dated to 26,913–28,348 yr cal BP [24]. The age of the artwork was determined both indirectly by chrono-stratigraphic association and by obtaining an Accelerator Mass Spectrometry radiocarbon (AMS ^14^C) age on ash adhering to the painted stone’s back surface [31].

In the Kimberley, geochronologist, Alan Watchman [21] obtained AMS ^14^C estimates from mineral encrustations covering paintings of the Irregular Infill Animal Period (IIAP) and Gwion Period, the two earliest painting styles identified in the relative regional sequence [14]. Analysis of samples from five sites on the lower Drysdale River showed that pigments used to produce the paintings did not contain organic material such as binders and therefore could not be directly dated [21]. Instead, samples for dating were obtained from mineral crusts lying as close to the paint layers as possible in order to determine minimum or maximum age estimates for the paintings [21]. Two minimum age estimates were obtained for Tassel Bradshaws (now known as Mambi Gwion–an earlier sub-group within the Gwion Period). Results produced minimum AMS ^14^C ages of 538–623 AD and 240–852 AD (both calibrated) for the accretion over a Mambi Gwion, but an older minimum age (2,559–2,149 B.C. calibrated) for the mineral skin over a Cane Bradshaw (now known as Gwion) considered to be a more recent Gwion sub-style in Walsh’s proposed sequence. As these are all minimum age estimates, and if Walsh’s relative stylistic sequence is accurate, the Mambi Gwion style would also predate the minimum AMS ^14^C age estimate of 2,559–2,149 B.C. obtained for the ‘Cane’ Gwion. A sample overlying an IIAP zoomorphic figure, the stylistic period thought to predate the Gwion Period, returned a minimum AMS ^14^C age estimate of 1,875–930 (B.C. calibrated). Watchman *et al*.’s [21] ‘preliminary results’ show that Gwion Period paintings are at least mid-Holocene in age.

An AMS ^14^C age of 3,780 ± 60 years BP (2,457–2,033 BCE calibrated) for a simple Wanjina head made from beeswax pellets provided a direct date for the emergence of the Wanjina Period [9]. ‘Classic’ Wanjina-style paintings appeared more recently, however, with ages for black pigments associated with such painting ranging from 1,210 ± 140 BP to modern [9,20] dated organics embedded within mud-dauber wasp nests overlying a Wanjina Period motif, and obtained a minimum AMS ^14^C age estimate of 990 ± 60 BP for the oldest sample [24].

The divergence of these findings illustrate the difficulties arising when chronologies are based on just a few minimum ages as these date the timing of the development of accretions or nests over the art only, and do not account for the potential time-lag between the painting or engraving episode and the formation of the skin or nest [23]. The relationship between the age estimates obtained for overlying nest, crust or skin and the creation of the image may not be closely aligned [32]. Therefore, it will be essential to build an extensive database of minimum (and maximum) age estimates before a realistic temporal framework for the Kimberley rock art sequence can be established.

**Fig 1:**
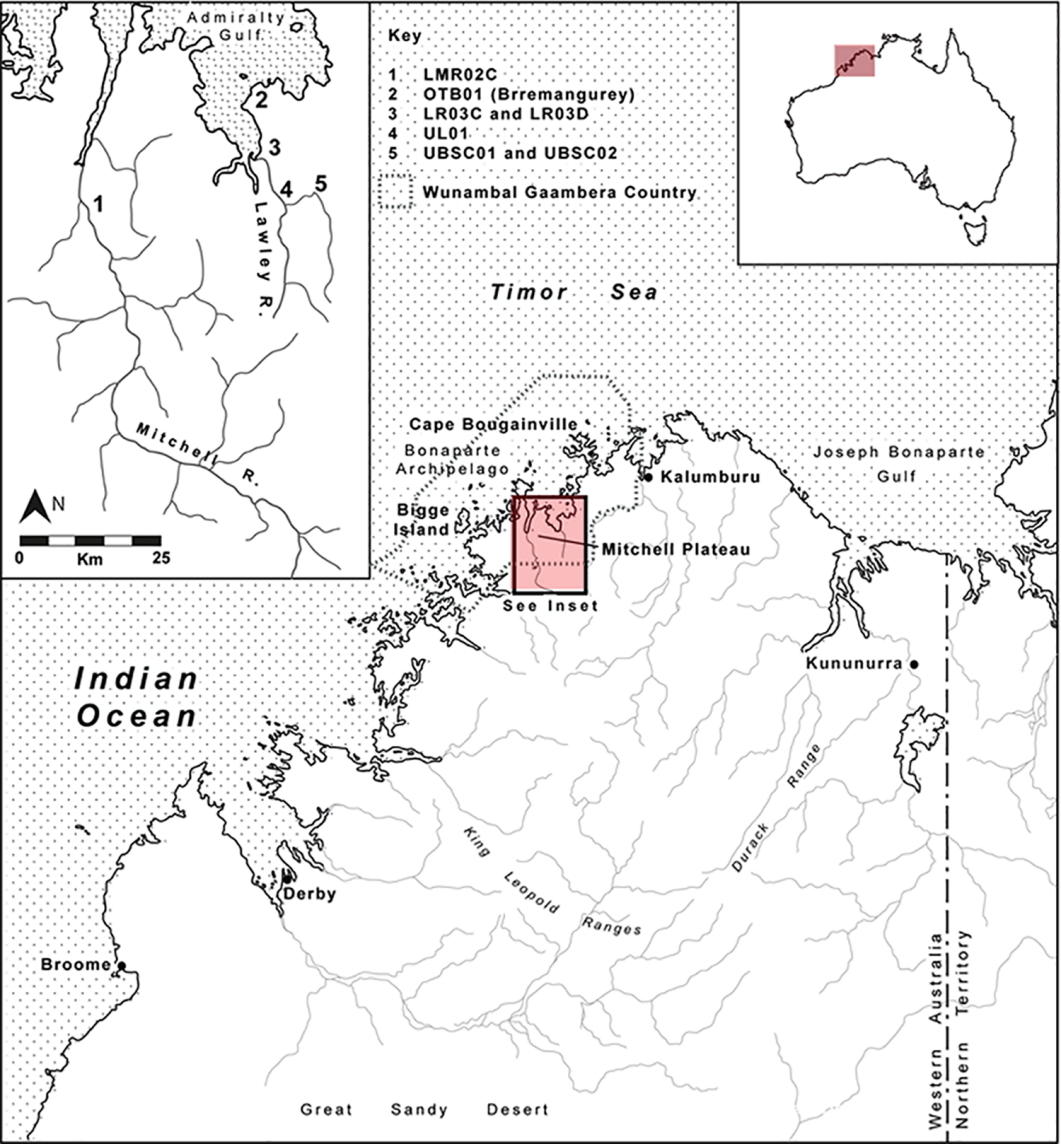
Mitchell and Lawley River drainage basins, northwest Kimberley, Australia.

With this as a major aim, a rock art dating project was undertaken to apply independent dating techniques to different Kimberley rock art styles. The research forms part of a much broader archaeological research project: *Change and Continuity*: *archaeology*, *chronology and art in the northwest Kimberley*, *Australia* focused on the Mitchell and Lawley River drainage basins (Fig 1).

## Background

The research has been undertaken with agreement and participation of the Wunambal Gaambera people, who are the recently determined Uunguu native title holders for Wunambal Gaambera country; their Wanjina Wunggurr culture is also listed as part of the West Kimberley included on the National Heritage List. The story that researchers are building sits alongside Wunambal Gaambera people’s Wanjina Wunggurr culture and religious beliefs, which have been integrated into an understanding and exploration of the material evidence in order to supplement the Wanjina Wunggurr traditions and their cultural world view. With modern research techniques, results can assist Traditional Owners to gain knowledge and insights into their ancestors’ life and existence and contribute to Wunambal Gaambera capacity to manage and keep their country and culture healthy. The Traditional Owners granted permission to obtain samples for dating from over the art, but requested that pigment was not removed in the process. Therefore, samples from under the art were not collected for analysis.

### Mudwasps

**Fig 2:**
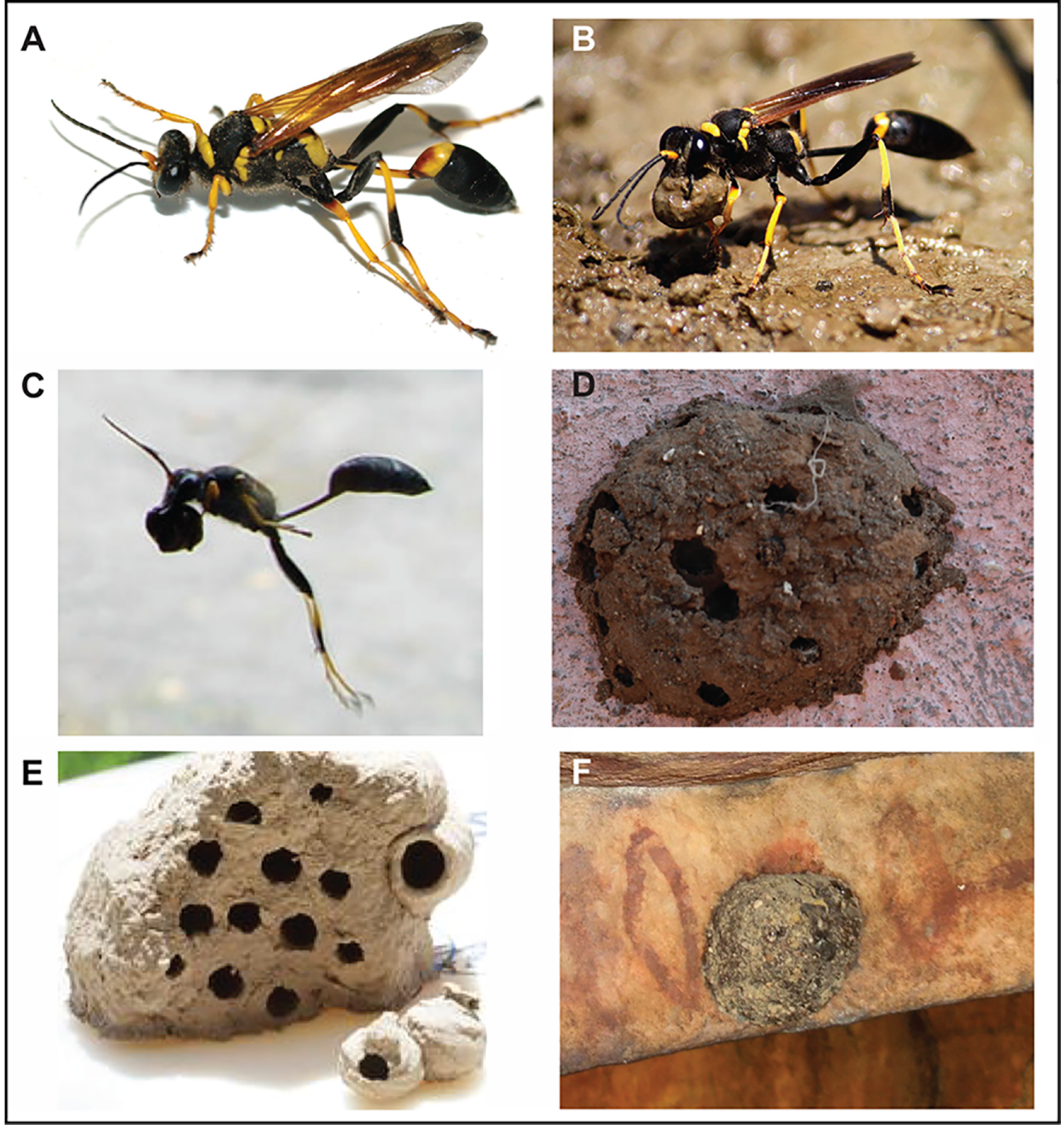
The black and yellow mudwasp (*Sceliphon laetum*) commonly found in the Kimberley region and their associated mud nests. **This wasp creates single-urn shaped cells that are joined by successive nest building to form a series of cells that are smoothed with mud on the outside to form one larger nest. Some wasp are primary nest builders on the bare surface of rock but most are secondary builders in that they build the nest on the stub of a former nest.** (A) the wasp (B) collects a ball of sediment and (C) flies holding the ball–this is most probably when most of the sediment bleaching occurs, (D) a fresh nest of single cells that has been smoothed with an outer layer of sediment (E) an older nest that has dried out and (F) a fossilised nest in situ and on-art.

**Fig 3:**
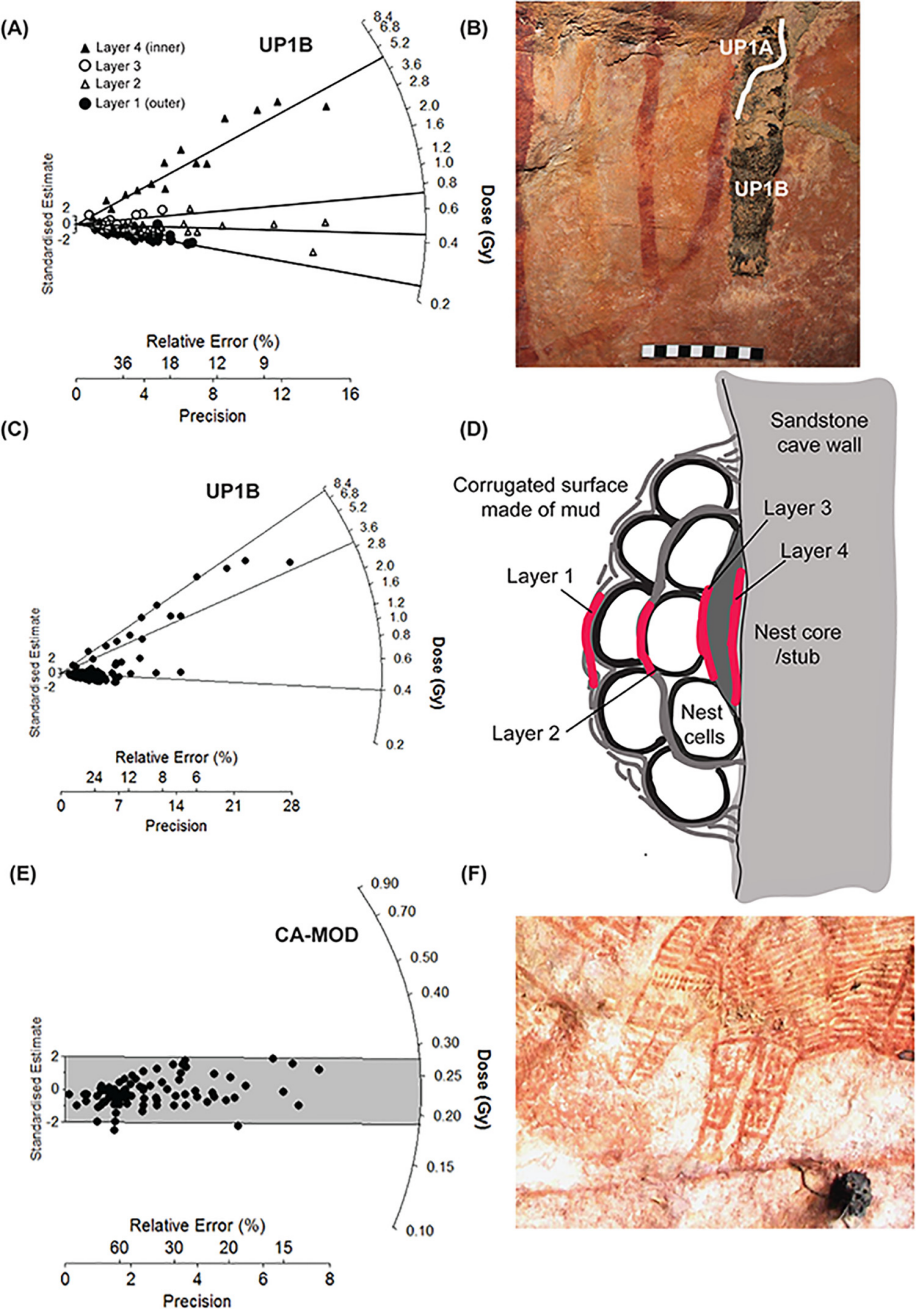
**Bleaching tests and results for the off-art nests from a modern sample (CA-MOD, A-D) and an ancient sample (UP1B, E-F).** (A) a combined radial plot of the 4 separate layers measured within the nest to demonstrate how the grain populations increase with distance towards the core. (B) The large nest sampled in the Upper Lawley region–the upper on-art section (UP1A) was used for dating while the lower off-art section was used for bleaching tests. (C) when the D_e_ values for all the layes are combined and analysed using the FMM, three dose populations have been determined and plotted on this radial plot. (D) a sketch of the mudwasp nest construction in relation to the sampling procedure for the bleaching tests. (E) A radial plot of the accepted grains from the entire modern nest (CA-MOD), the majority of grains lie within 2 sigma of the central age–as determined by the CAM at 0.23 ± 0.1 Gy, which at at dose rate of 1.11 ± 0.5 Gy/ka^-1^ represents an age of only 220 ± 10 yrs. (F) The location of the modern off-art nest CA-MOD in LR02 situated close to the CA-7 nest and Argula motif.

Mud dauber (sometimes called “dirt dauber”, “dirt digger”, “dirt dobber”, “dirt diver”, or “mudwasp”) is a name commonly applied to a number of wasps from either the family *Sphecidae* or *Crabronidae* that build their nests from mud. Most common to this region of the Kimberley is black and yellow mud dauber wasp (*Sceliphon laetum*) [33–34] that are long, slender wasps about 1-inch (25 mm) in length (Fig 2A–2C) that are common in tropical to sub-tropical sandstone areas [35]. Their name is derived from their distinctive nests moulded into place by the female wasp’s mandibles, which take the form of a simple, sausage-shaped cells containing one egg that are clumped together to make a cluster of parallel cells arranged with their long axes horizontal (Fig 3D). This cluster is plastered over to form a smooth nest and developed into a series of mud corrugations that fall off the older nests [33] (Fig 2D–2F). The wasps collect material to make the nests from the surrounding landscape from 25–200 m away [33] using surface sediments so it contains sand-sized quartz and feldspar, pollen and other organics. It is these nests, constructed in crevices, cracks and other shielded locations like rockshelters, which are sometimes found in a fossilised state (Fig 2F) over and under rock art motifs thus providing potential material for dating. This species may occupy the same sites year after year creating large numbers of nests that can remain *in situ* in protected locations for extended periods of time. Usually, the wasp favour the stub of a former nest to construct their new nests [33], thus at least two phases of nest formation can be observed in each nest [20,36].

### Establishing a chronology for Mudwasp nests using optically-stimulated luminescence (OSL) dating

OSL is a light sensitive signal that builds up over time during a period of ‘burial’ or cover. Provided the samples are not exposed to light during burial or collection, the signal can be stimulated in laboratory conditions and measured. When divided by the natural radioactivity of the soil or substrate, the amount of light (luminescence) produced is proportional to the period of burial time. OSL is the main method used for establishing chronologies for excavated occupation deposits that pre-date the maximum AMS ^14^C boundary, or in deposits that lack ample carbon samples. Samples of sediment found within the cave environment have no direct association with the art, but sediments may be collected by wasps and then ‘buried’ within mudwasp nests found on top of the art. Dating of mudwasp nests using OSL was first introduced by [20]. They initially worked with large nests and sampled each layer to determine the extent to which the light no longer penetrated the nests and the quartz was effectively ‘buried’ and supported their OSL age estimates with AMS ^14^C dating of organics found within two nests [20].

In their pioneering work, Roberts *et al*. [20] recognised that in certain circumstances the paucity of large nests suitable for dating necessitated the used the entire nest to maximise the number of quartz grains used for dating. However, the procedure was further developed by Yoshida *et al*. [37] using linearly modulated OSL (LM-OSL) where the power of the stimulating laser is increased slowly during measurement as opposed to the traditionally used continuous wave (CW) stimulation. This allows the ‘fast’ component of the OSL signal (that is easily removed by sunlight or ‘bleaches’ rapidly) to be distinguished from the slow component (that bleaches very slowly). The differences between the two signal components can provide evidence for the degree of bleaching received by each grain prior to burial, thus they retrospectively identified the ‘light-safe’ grains. Aubert [22] in his review of rock art dating recommended that to obtain robust age estimates using the mudwasp nest technique: 1) the relationship between the quartz grains and the art must be clearly established, 2) phases of nest development are established with a consideration of the nest stub, and 3) only large nests are sampled.

## Methods

Research was completed under Western Australian Department of Indigenous Affairs, Section 16 Permit Nos. 465 (2010), 490 (2011–12), and Authority 4 Permits from the Western Australian Department of Environment and Conservation (DEC), CE002829 (2010), CE003254 (2011), and CE003574 (2012).

In order to develop a robust regional chronology for the art, we trialled three different dating techniques to act as independent age estimates. 1) OSL dating was applied to quartz grains in mudwasp nests, 2) AMS ^14^C dating was applied to beeswax resin deposited over art and for organic material found in the matrix of mudwasp nests, and 3) Uranium Series (U/Th) was applied to thin veneers of silica and to precipitated gypsum crusts that had formed over motifs. Our aim was to target classic examples of each of the major stylistic periods and compare results from each of the three techniques as a means of confirming the integrity of the age estimates obtained.

### Optically-stimulated luminescence dating

#### Nest sampling

Sites where suitable dating opportunities existed were selected from the rock art sites recorded along the Mitchell and Lawley Rivers during the previous years of the project. Despite the range of rock art sites recorded (204), identifying suitable samples for dating proved more difficult than we had anticipated but, despite the challenges presented by the rugged terrain (sites have to be accessed by helicopter or boat), the thinness of some of the accretions, and the necessity of collecting OSL samples in low light conditions, 11 samples (from *Sphecidae* family) were collected for processing. All nine on-art samples were located directly on top of rock art thus avoiding problems of association and to ensure that all phases of nest construction are used to constrain the age of the art.

At the request of the Traditional Owners, and with protection of the art as a major consideration, all mudwasp nests sampled were located *on top* of rock art so that the resulting age estimates represent minimum ages only. These provide a useful baseline for the development of a chronological sequence but they do not provide an absolute date for the underlying art, neither do they establish an upper age limit.

The limited sampling situation and the absence of large nests on-art meant that we had to take the opportunity to use the entire nest in specific cases (where they overlay distinctive rock art motifs) rather than using the more elusive but ideal larger nests. In these cases, we adopted the all-nest sampling technique proposed by Yoshida *et al*. [37].

Despite extensive fieldwork in the region, the large fossilised nests identified as ‘ideal’ by Aubert [22] were not located on top of art. Instead, we found and sampled nine smaller nests, typically 15 x 15 mm in width, with a thickness of between 5–20 mm and ~1g, yielding between 1–10 mg of quartz each. One of the nests (UP1A) was larger with ~20 mg of quartz obtained, so this was used for testing of the luminescence procedures, further analyses and dosimetry measurements. Seven of the nests were located wholly on top of the art. In these situations the relationship between the quartz grains comprising the nest and art below is very clear [22] thus ensuring that results provide a minimum age estimate for the production of the underlying art. This is important as even if the nest was extended on a nest stub by wasps at a later date, both the older stub and younger overlying nest provide a minimum age for the art. The two remaining nests, UP1A and BRY-6 extended off art with ~50% of the nest on the surrounding rock. In these cases, we only sampled the section of the nest that was located directly on top of the art so that uncertainties about the relationship between the art and the nest were eliminated.

The mudwasp nests were carefully removed from the rock surface using a small razor blade in subdued red light conditions. Quartz grains of 180–212 μm diameter were separated from the matrix under dim red illumination using standard purification procedures, including a final etch in 40% hydrofluoric acid for 45 minutes to remove the external alpha-dosed rinds [38]. The very low quartz yield meant that for some samples certain procedures were modified such as removing the 2.62 mg separation to maximize the amount of material prior to a hydrofluoric acid (HF) etching procedure. The feldspars were then removed during the HF procedure and the presence of feldspar contamination was checked prior to measurement using an IR wash and after measurement using a light microscope.

#### Stimulation and testing of the quartz grains

We used OSL techniques applied to single-grains of quartz incorporating a modified SAR protocol [39] for between 100–500 single-grains loaded on aluminum single-grain discs and measured in a TL-DA-20 Risø unit containing the single-grain attachment [40–41]. Each of the quartz grains were stimulated for 2 s using a 10 mW 532 nm Nd:YV0_4_ solid-state diode-pumped green laser with 50% power corresponding to 25 W/cm^2^ [41–42], and the ultraviolet emissions were detected by an Electron Tubes Ltd 9235QA photomultiplier tube fitted with 7.5 mm of Hoya U-340 filter. All of the available grains from the entire nest were used for the SG-OSL dating procedure rather than just the core. The percentage of grains that emitted luminescence was high (typically 20–30%), with only one sample (BRY-6) with a high rejection rate (91%). As this provided a less than ideal minimum number of quartz grains on which to base the results, we have flagged this in our results. All of the grains from the small nests were measured with a continuous laser power (CW). In addition, we adopted Robert *et al*.’s [20] techniques for the larger nests (sample UP1A and the two off-art test nests UP1B and CA-MOD) using linearly-modulated OSL measurements (LM-OSL) to isolate the bleached outer grains and the non-bleached inner grains. For LM-OSL measurements the laser power was increased from 0 to 90% over a 30 s period at 125°C corresponding to 0–45 W/cm^2^ of power.

The only nest that contained enough quartz to conduct tests on the luminescence characteristics was UP1A so preheat plateau and dose recovery tests were performed on this sample. Small 1 mm mask aliquots of the fresh quartz (180–212 um size fraction) were prepared containing only a few hundred grains, and a SAR run was conducted on three aliquots at each of the preheat temperatures tested; 200, 210, 220, 230, 240, 250, 260, 270°C for 10 s (a total of 24 discs). When plotted, this revealed a preheat plateau at ~250°C so this temperature was chosen to perform dose recovery tests. The natural OSL signal was bleached using 100 s of blue diodes at 50°C temperature and a SAR run was conducted on 8 aliquots using a surrogate dose of 20 Gy. All 8 aliquots returned a dose within 2 sigma error of the surrogate dose confirming that the measurement protocol was valid, the recycling ratios were low and there was negligible recuperation (Fig 2).

#### Estimating the environmental dose rate

To estimate the environmental dose rate at the sites *in situ* measurements were made using a Canberra Inspector 1000 portable gamma spectrometer. To assess the gamma contribution from just the sandstone bedrock it was impractical to create holes large enough to accommodate the 2-inch gamma spectrometer probe to allow a 4 π geometry around the scintillation crystal. More importantly, the holes would have had to be created in the art itself, which goes against the principles of this project. Instead, the probe was held flush against the sandstone rock surface during measuring. This provided a measurement of the contribution from the surrounding rock shelter but not an accurate measurement from the bedrock itself. This count, as a 2π geometry, was then compared to counts derived from a technique used to determine a 4 geometry without burying the probe [43]. Therefore a comparison could be made between the contribution from the rock alone verses the contribution from a 30 cm hemisphere around the location of the mud wasp nest.

The samples were divided into motifs that were located on horizontal panels (roof of the rockshelter) with no other rockshelter walls or floor within at least 1.5 m and motifs that were located on vertical panels or horizontal panels close to the cave floor. For the former group, the gamma spectrometry measurement provides a true 2π geometry as there are no other source of gamma rays to contribute, thus the measurement was doubled to create a 4π geometry and the equivalent of a probe buried in sandstone. For the second group, we isolated the gamma ray contribution from the other walls and floor by taking an additional measurement but this time with a 20 cm radius and 4 cm thick polymethyl methacrylate (Perspex) screen to act as an attenuator, which lay flat against the rock surface on one side and the probe on the other side [43]. As Perspex reduces gamma rays the contribution from the rock itself is minimized and the counts reflect the gamma contribution from the cave floor and surrounding walls. Thus, the data from the Perspex measurement is subtracted from the original measurement to estimation the gamma ray contribution from only the intended rock surface. This is then the equivalent 2π geometry that is treated in the same manner to represent a 4π geometry.

In addition, concentrations of ^238^U, ^235^U, ^232^Th (and their decay products) and ^40^K were estimated using the count rate derived from a Geiger-Muller multi-counter for beta counting of dried and powdered sediment samples in the laboratory from a section of the mudwasp nest and small amount of the sandstone rock surface. Due to the rockshelter environment, cosmic dose contribution was estimated by taking into account sandstone shielding, rock geometry, altitude and latitude. To estimate water content a small section of the larger off-art nest (UP1B) was separated and dried to compare the wet weight verse the dry weight. In addition, we estimated the weight of its maximum saturation by adding water to a suspended sample until it began to release liquid. Thus within the known boundaries of dry-saturated and present day weights, we could more accurately estimate how the water content has changed over time. We did not detect any change in weight so assigned a water content of 0 ± 0% for all the nests sampled, apart from the CA-MOD nest which we assigned 2 ± 0.2%.

#### Disequilibrium in the mud nest and on the rock surface

Disequilibrium is a deficiency or excess in the daughter nuclides in relation to the parent nuclides. Excesses or deficiencies in the decay chains of ^238^U and ^232^Th can cause over and underestimations in the estimated dose rate that has a knock on effect on the final age estimation. Disequilibrium is known to occur in sediments and on rock surfaces, and in addition radon, a daughter nuclide in gaseous form, is present in sandstones so we tested for these possible effects. Due to the difficulty of establishing an accurate dose rate for the context of the nests on a sandstone surface, a section of one of the larger off-art nests (UP1B) and a small section of the recent off-art nest (CA-MOD) were sent off for high resolution gamma spectrometry (HRGS) to check for disequilibrium in the decay chains and to compare the dosimetry of a modern verses an ancient nest. This test could not be conducted for all samples due to a lack of material.

A final dosimetry consideration is the use of HRGS as opposed to Neutron activation analysis (NAA) for investigating the disequilibrium in the decay chains. Some radioisotopes such as ^232^Th, ^234^U and ^230^Th do not emit gamma rays so cannot be measured using the former technique. Therefore, we have made allowance for this potential loss and have factored this into the error margin.

#### Analysis of the single grains of quartz

Quartz SG-OSL analysis allows aberrant and non-luminescing grains to be removed from the equivalent dose (D_e_) determination and enables an assessment of the dose populations within one sample. This ensures that the grains that have been exposed to the light (on the outer sections of the nest) can be separated from the grains that have acquired a dose during burial (the core of the nest). In a processed sample, the grains will naturally contain a range of D_e_ values called ‘scatter’–in an ideal sample this observed scatter can be explained by a natural statistical variation or a Gaussian distribution, so that 95% of all D_e_ estimates lies within 2 standard errors [35]. However, many samples contain a much wider range of D_e_ values than a normal distribution and this is termed overdispersion and can be caused by; 1) older or younger grains intruding into the layer from post-depositional mixing or roof spall, 2) insufficient bleaching of the grains prior to burial and 3) differences in the microdosimetry influencing individual quartz grains. For the mudwasp nests the post depositional mixing within a sedimentary layer is not a problem as the grains are separated by the nest building process but contamination is a potential problem from the host bedrock, so when analyzing the SG-OSL data we look out for grains with an inherently large dose and a low sensitivity to luminescence [44–45]. We assumed that the second problem is not an issue due to the bleaching potential of the sampling, flight and nest building processes of the mudwasp (bleaching tests are outlined in the next section), while the third could be a potential problem that is investigated in the dosimetry section. Grains with undesirable luminescence characteristics due to contamination or natural occurrence have been rejected according to the procedure outlined in Jacobs *et al*. [46–47].

The measurement of single-grains invariably reveals a variation in the precision of each D_e_ value as some grains are inherently much brighter than others, so grains with a similar D_e_ value can have a precision of <5% for very bright grains and >50% for dim grains. To incorporate this information into D_e_ determination a radial plot is used [48]. Therefore, the D_e_ value of the grain (read from the left hand axis through the point to the intersect with the right hand dose axis) can been observed along with its precision (read from the point vertically down to the precision axis). Using the overdisperson values for the sample combined with the distribution seen in the radial plot allows an estimation of whether the data represents a single or multiple populations. A population is defined as a group of grains with D_e_ values and errors that can be described by a Gaussian distribution and that are statistically outside the range of another distribution. As more than one population has been interpreted in the mudwasp data, we have analysed the grains using a Finite Mixture Model (FMM) [49]. This statistical analysis assumes that there is more than one dose population within the grain distribution and aims to identify the number, D_e_ values and overdispersion of each population.

#### The bleaching and burial of grains in the nests

The assumption of this technique is that the quartz grains were all well-bleached before being buried in the nest so that any signal that accumulates is derived from radioactive nuclides in the nest and on the rock surface rather than from a residual OSL signal. It was also assumed that, even for the small nests, there is sufficient light shielding for the inner core grains to accumulate a signal that can be measured. Therefore, the differences in the OSL signal between grains in a nest results from differential exposure of grains to light during nest construction, that is, the outer grains are bleached due to light exposure, the middle grains are semi-bleached due to some exposure before being covered, while the inner grains should retain the largest signal due to longest ‘burial’ period. However, Roberts *et al*. [36] found that even grains found close to the surface of their nests still retained a small residual dose, which they attributed to the timing of mud sampling in freshly exposed sediment. In addition, some of the nests retained their outer coating of mud, but for some, this layer has fallen away either during the fossilization process, or when the young wasps exited the cells. It is therefore important to determine the difference in signal retention between the nest with and without the original crust. To test these assumptions at our sites and to establish a baseline of bleaching we conducted bleaching tests. As only small nests were discovered, all of the available nest material was used for sample processing so testing this assumption on these samples proved difficult. Instead we used SG-OSL techniques applied to off-art nests by:

1. Measuring bleaching of the grains within a modern nest (sample CA-MOD 20 x 20 mm). This nest was determined to be modern as it was still fresh (wet) with no fossilization and the team could verify that it was not present at the time of the previous year’s fieldtrip so its age is < 1 yr. The nest still retains its original outer coating. In theory, the grains should only contain very small OSL signals; larger OSL signals may indicate that the grains are retaining a residual dose prior to being buried in the nest (Fig 3E and 3F)
2. Measuring and comparing the difference in signal between the grains that form the inner and outer parts of a large (65 x 20 mm) ancient nest (sample UP1B) by sampling each layer within the nest for OSL dating. This sample represents the bottom half of the UP1A sample used for dating, but this section is much larger and lies off-art. In addition, the outer layer of the nest is absent in places so this provides a useful test of signal retention (Fig 3B). In theory, there should be a gradation of signal with the inner core containing the most signal and the outer grains containing the least.

Before sampling the nests, we considered their construction and taphonomic history by observation and using footage of nest construction. It was determined that the nests have a core that is a residual stub of a former nest, and each cylindrical tube is built separately on the base core and then joined with mud. Therefore, we sampled the ancient nest in four layers. The outer layer (L1) comprised the outer 1–2 mm of the nest, then the next two layers (L2-L3) comprised of mud from successive layers towards the core, and finally the fourth layer (L4) comprised of the nest core or stub (Fig 3D). A comparison of the layers was used to test for initial bleaching, signal accumulation and bleaching of the outer layer, while for the modern sample we used the entire nest to test the distribution of doses within the grains. Due to the large size of these nests, we were able to measure single-grains using both CW-OSL and LM-OSL laser measurements for both the modern and ancient nests. The first 5 s of the 30 stimulation LM-OSL measurements were used to represent the fast component, while the last 5 s were used as the slow component with a blank disc used to provide a background measurement. The D_e_ values derived were then divided to provide a fast:slow ratio (f:s).

The results of these tests (see results) justified the bleaching assumptions, the use of the entire nest for sample processing and the use of a finite mixture model (FMM) to analyse the SG dose populations [49]. Therefore, in this context it has been used to isolate the bleached outer from the semi-bleached inner and non-bleached core of the nest. Prior to running this model, a 10% overdispersion value was added to the standard error as an estimate of the inherent overdispersion within the grains. This value was determined by bleaching fresh aliquots of sample UP1B using a solar lamp (Philips MLU 300W mercury-discharge sun lamp) for four hours and measuring the overdispersion between the resulting D_e_ values.

Where possible we compared the OSL results from mudwasp nests with AMS ^14^C dating of either beeswax resin found on the art of the same motif or from AMS ^14^C dating of pollen from the actual mudwasp nest. Due to the limited amount of material available for OSL dating we used pollen found in the <90 um size fraction that is usually not used for OSL dating. Only one sample (BRY-6) yielded enough material from this size fraction to be processed by ^14^C.

### Accelerator Mass Spectrometry radiocarbon dating

The material sampled for AMS ^14^C dating is a resinous compound originating from native stingless bees rather than pure wax. Australia has around 12 species of native stingless bees, three of which are found in the Kimberley and recognised by Wunambal Gaambera people [50–51]. These three species are divided into two genera: *Austroplebeia* and *Trigona*; the latter comprises two distinct species within the genus: *Tetragonula hockingsi* and *T*. *mellipes* (*Tetragonula* is a subgenera of the genus *Trigona*) [51–52].

Native stingless bees are small (about 3–5 mm in length), compact, black bees and, as the name suggests, have no sting. They build their nests in hollow tree trunks, branches, fallen logs, or in termite mounds or between and under sandstone boulders, in rock crevices, or on cliff faces [50–51,53]. Their nests are made of wax and a resinous compound. These elements are likely to incorporate aggregations of pollen grains, pieces of young adult bee exoskeleton and fragments of xylem fibres and vessels [54] making them ideal for AMS ^14^C dating. Samples were collected in a similar manner to those obtained for OSL dating although all were collected during the day. Standard AMS ^14^C dating techniques were using to process the samples.

### Uranium Series dating of rock surface coatings

Twenty-three samples of gypsum rich coatings and amorphous silica skins were collected for U-series dating. The results yielded either insufficient U-series isotopes for dating or proved to be too contaminated with detrital material to provide a meaningful age estimate. Therefore no comparisons could be made using this technique. (M. Aubert, pers. comm.)

## The Rock Art and Samples Selected for Dating

**Fig 4:**
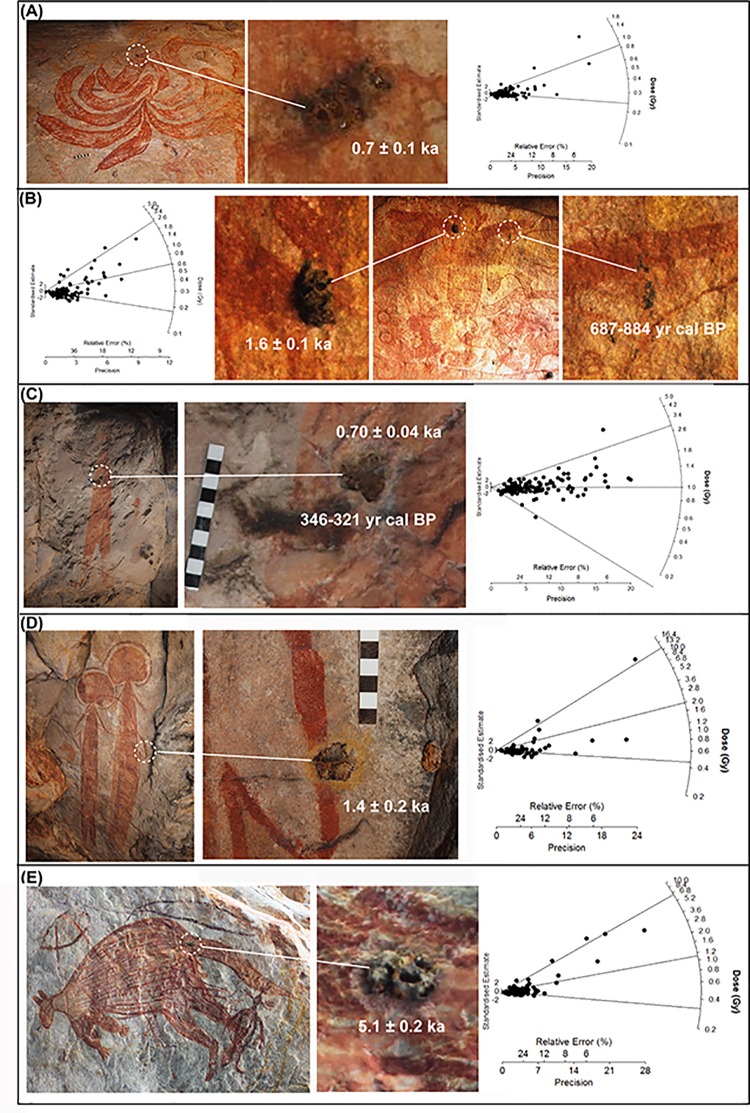
Wanjina Period rock art. (A) JS-11, ‘star-yam’ form, sampled mudwasp nest is indicated and enlarged. The resulting radial plot is also shown with a D_e_ according to the FMM 0.85 ± 0.15 Gy. (B) CA-7, Wanjina Period ‘*Argula*’ figure with sampled mudwasp nest for OSL dating (circled left and enlarged) and sampled beeswax resin for ^14^C (circled right and enlarged). This motif offered an opportunity for a comparison between OSL and ^14^C dating. The resulting radial plot is shown on the left with a D_e_ according to the FMM of 2.49 ± 0.13 Gy, while the ^14^C returned an age of 687–884 yrs cal BP. (C) BRY-3, Wanjina Period large red anthropomorphic figure, sampled mudwasp nest circled and enlarged, scale is 10 cm. Radial plot fitted with FMM provides a D_e_ of 0.99 ± 0.04 Gy, while the ^14^C returned a minimum age estimate of 346–321 yrs cal BP (D) BRY-6, Wanjina Period fish superimposing two anthropomorphic figures, sampled mudwasp nest circled and enlarged. Radial plot fitted with FMM provides a D_e_ of 1.97 ± 0.72 Gy. (E) LM-13 Wanjina Period macropod, sampled mudwasp nest circled and enlarged, photograph digitally enhanced using D-Stretch. Radial plot fitted with FMM provides a D_e_ of 6.44 ± 0.12 Gy.

Fig 4A. A large motif painted in a ‘star-yam’ form was located on the back wall of a large rockshelter (UBSC02) at the headwaters of Bush Spirit Creek. The shelter contains a substantial body of rock art with most phases of the Kimberley stylistic sequence represented. The red/orange motif is infilled with roughly painted dashed lines. A small (15 x 5 mm, JS-11) circular mudwasp nest with a hard outer coating that has been partially eroded was located on the perimeter of the motif.

Fig 4B. An anthropomorphic figure (280 mm high) with raised legs, large ears and exaggerated male genitalia is painted on a vertical white wall at the rear of the large cavern (LR03D) and was identified by Traditional Owners as an *Argula* (S1 Fig). *Argulas* are one of a number of spirit figures that still play a role in the belief system of the local Wunambal Gaambera people today [5,8]. A small oval nest (30 x 20 mm, CA-7) comprised of three mudwasp nest tubes with an intact fossilised outer covering is located on the left shoulder of the motif.

Fig 4C. Two motifs were sampled from Brremangurey (OTB01), a large rockshelter adjacent to the shoreline on Admiralty Gulf, which contains an extensive body of rock art representing all stylistic periods (S2 Fig). The first, a large red anthropomorphic figure (1380 mm high) located on the ceiling in the centre of the shelter. The motif is depicted with a ‘shocked’ headdress, in full frontal position, with out-turned feet, and sloping arms with two-fingered hands. A small circular nest (15 x 15 mm, BRY-3) with a fossilised hard outer coating is located in the centre of the body.

Fig 4D. The second motif from Brremangurey, a bichrome fish painted on the rear ceiling overlies two polychrome anthropomorphic figures (both 2,500 mm high). These are depicted with halo headdresses, three-fingered hands and turned out feet. The overlying fish is painted in plan perspective and has outlined eyes, double gill bands and a row of ordered short-lined infill. A small round nest (15 x 15 mm, BRY-6) with a fossilised outer coating covering two mudwasp nest tubes partially covers the fin of the fish.

Fig 4E. A large macropod motif painted in polychrome (yellow, red and white) is located on the ceiling of a large overhang (LMR02C) that forms part of a cluster of art sites east of the Lower Mitchell River Falls (S3 Fig). The site contains an extensive body of rock art, including ‘classic’ Wanjina figures and 21 animal depictions that can be attributed to the Wanjina Period. Both the body and limbs of the macropod are depicted in profile and are divided into sections at the neck, shoulder, elbow and foot. Both eyes and ears are shown with a twisted perspective. The macropod is depicted with a central backbone and kidneys in a manner similar to the X-ray art of Arnhem Land. The entire motif is infilled with fine longitudinal lines, while white dotting has been added to the shoulders, forearms and kidneys. A small, round, well-fossilised mudwasp nest with a partial outer covering (20 x 25 mm, LM-13) is located on the upper section of its tail.

**Fig 5:**
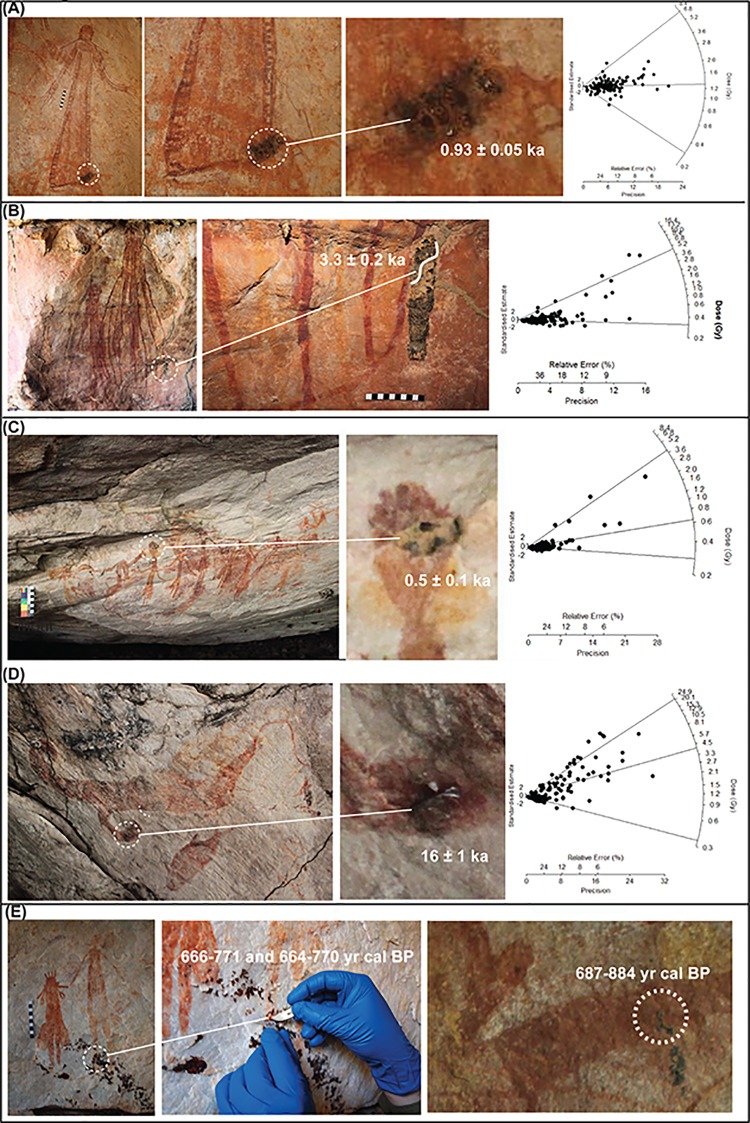
Gwion Period rock art. (A) JS-10—Anthropomorphic figure with a distinctive triangular ‘robe’ shaped body, sampled mudwasp nest circled, scale is 10 cm. Radial plot fitted with FMM provides a D_e_ of 1.32 ± 0.04 Gy. (B) UP1A—A pair of Mambi Gwion (Tassel Bradshaw) anthropomorphic figures superimposed by a ‘cage-shape’ motif, sampled mudwasp nest circled and enlarged. Note only the upper section of the nest (UP1A) was sampled for dating, the lower section (UP1B) was used for bleaching tests as an off-art ancient nest. Radial plot for UP1A fitted with FMM provides a D_e_ of 4.61 ± 0.12 Gy. (C) CA-9—Horizontal composition of transitionary Gwion/Wararrajai Gwion Period anthropomorphic figures, sampled mudwasp nest circled and enlarged, scale is 10 cm. Radial plot fitted with FMM provides a D_e_ of 0.63 ± 0.11 Gy. (D) CA-8, an elongated ‘yam-like’ motif with a bifurcated ‘root’ or ‘tail’ with location of sampled mudwasp nest circled and enlarged. Radial plot for CA-8 fitted with FMM provides a D_e_ of 21 ± 1Gy. **(**E) LRO1C-2, LRO1C-3—Wararrajai Gwion Period anthropomorphic figure (on left) and LR03S-01—Wanjina Period ‘*Argula*’ figure (right) both sampled for beeswax resin (circled), scale is 10 cm. These resin samples provided AMS ^14^C age estimates of 687–884 yr cal BP and 666–771 yr cal BP, and 664–770 yr cal BP respectively.

**Fig 6:**
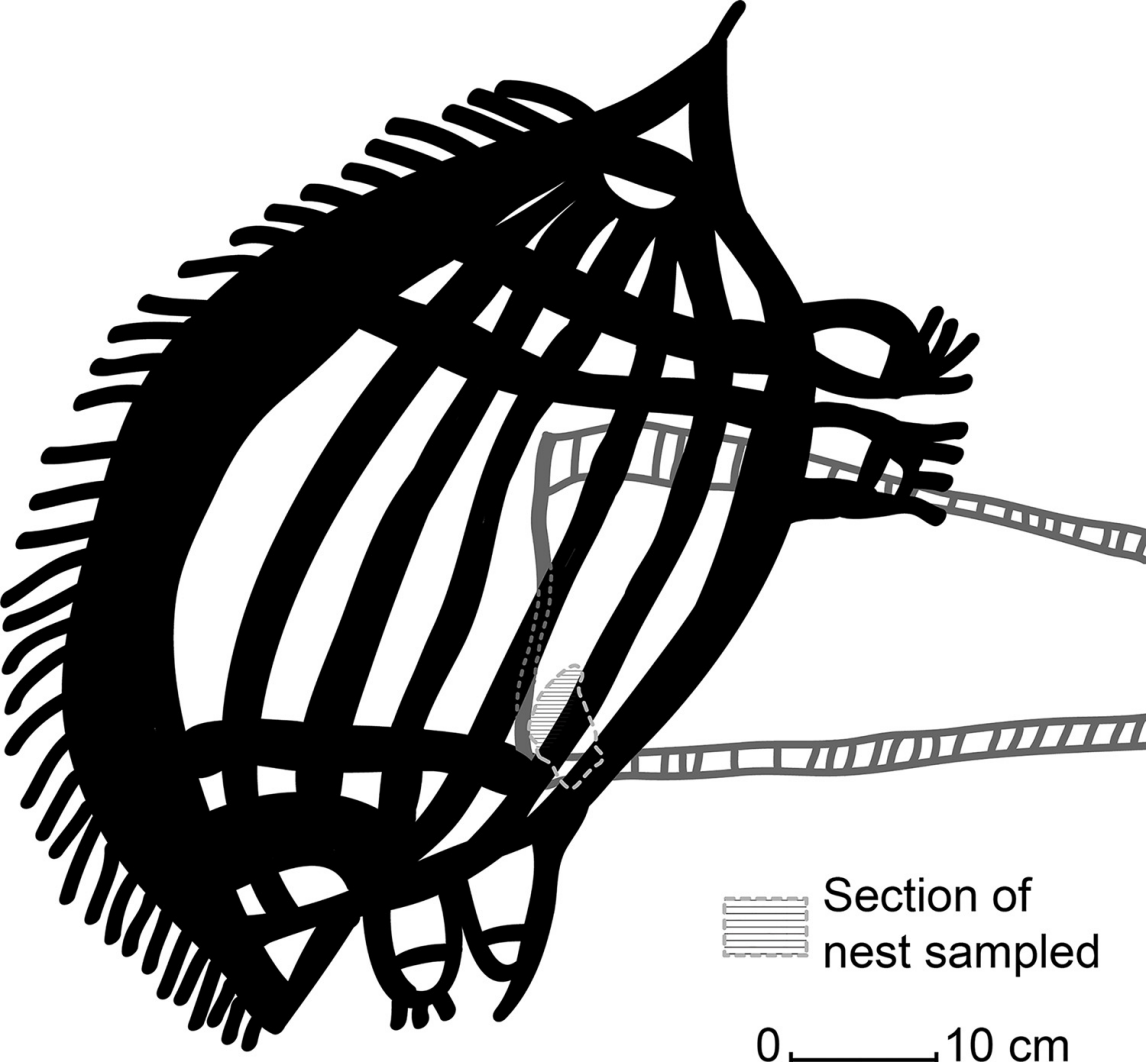
Echidna motif overlying sampled nest, which overlies the anthropomorphic figure with a distinctive triangular ‘robe’ shaped body.

Fig 5A. A large (910 x 500 mm) anthropomorphic figure with a distinctive triangular ‘robe’ shaped body with a dome-shaped hatched headdress and shoulder spikes was recorded at a site on the slope above Bush Spirit Creek (UBSC01). The dark red and orange figure is painted on the ceiling of a small overhang and is partially covered by a washy orange echidna motif (Fig 6). The echidna is painted in profile with thick outline and segmented body. An oval nest (30 x 15 mm area sampled, JS-10) with a partially removed top section was located on the hemline of the figure’s ‘robe’. The echidna motif covers the lower edge of the nest. This section was left *in situ*. A red hand stencil underlies the anthropomorphic figure.

Fig 5B. A pair of large, Mambi Gwion anthropomorphic figures (1,240 mm high) is located on a vertical panel that forms the wall of a narrow passageway through a rocky outlier (UL01) adjacent to the Lawley River. The figures are painted with red pigment and have a distinct, darker red outline. They include features such as tassels attached to the head, clearly defined fingers, paunch, narrow chest and hunched shoulders with decorative adornments at wrists and elbows They are superimposed by a red and yellow ‘cage-shape’ motif that outlines and, in turn overlies the larger of the Gwion figures. The top half (45 mm) of an elongated nest (110 x 20 mm, UP1A) is situated directly on top of the outer cage. The section sampled as UP1A retains a hard fossilised outer coating, but the lower section sampled for bleaching tests has a partially absent outer coating.

Fig 5C. A horizontal composition of ten, small transitionary Gwion/Wararrajai Gwion Period anthropomorphic figures is located in the same cavern (LR03D) as the *Argula* figure (Fig 4B) and the ‘yam-like’ motif discussed below (Fig 5D). The cavern forms part of an extensive network of underground caves, several of which contain rich assemblages of rock art. The complex is located under a rocky outlier, adjacent to a permanent water hole on a tributary that runs into the Lawley River as it approaches the shores of Admiralty Gulf. The composition of the figures into a frieze suggests a single painting episode although two distinct coloured pigments (dark red and red/orange) have been used. Some figures are depicted with paired boomerangs, bent knees and drooped dunce cap headdresses, while others have bodies formed by parallel lines, have straight legs and are shown in full frontal position. Similar friezes of small anthropomorphic motifs have been recorded at other classic Gwion sites. A small oval nest (10 x 5 mm, CA-9) located over the head of the central figure is comprised of just one or two mudwasp nest tubes wedged in against a fissure in the rock surface. It has lost all of its outer coating.

Fig 5D. An elongated ‘yam-like’ motif with a bifurcated ‘root’ or ‘tail’ is painted on a low section of the ceiling of the same large cavern (LR03D). The motif (1,200 x 1,100 mm) is painted in mulberry coloured pigment, typical of the colour of many of the older style motifs found across Australia’s northern regions [55]. However, at one end of the motif, the pigment has weathered to a deep red. While the extremities of the motif are solidly filled, the infill across most of the motif is best described as dense and irregular. The small round mudwasp nest (20 x 20 mm, CA-8) is located directly on top of one of the curved extremities. The nest still retains all of its outer coating and displayed the greatest amount of nest fossilisation of all the nests sampled.

Fig 5E. Three samples of beeswax resin (LR03C-2, LR03C-3, and LR03S-01) were collected overlying motifs located in the same complex of caverns (LR03). One sample, (LR03S-01) overlay the *Argula* motif (Fig 4B) described above, while the remaining two samples covered the legs of one of a pair of red anthropomorphic figures painted on the vertical wall of the adjacent cavern (LR03C). The figure is classified as a Wararrajai Gwion and is depicted in full frontal position with an outlined torso infilled with irregular lines, stick arms, a solid head, and legs that end in squared extremities and a headdress that sweeps off to the side.

## Results

The radial plots have been presented in Fig 4 and Fig 5, and D_e_ values using the CW FMM have been presented in Table 1. These results showed a wide spread of minimum age estimates for art motifs ranging from 530 years up to 16 ka. These age estimates have been determined using between 56–170 accepted grains For each sample, the FMM determined between 2–3 dose populations, the lowest of these (the lower population) correlated with the baseline of bleaching provided by the analysis of the modern nest, while the middle (if present) lies in between, and upper population contains the grains with the largest D_e_ values and was subsequently used for D_e_ estimation with between 15–25 grains being used for the upper dose population. As we are dealing with low grain number we have defined a population as statistically significant if it contains ~10% of the accepted grains, and as most of samples have between 56–170 accepted grains this represents ~6–17 grains. A statistically significant level i.e. the values reflect the characteristics of the population rather than just sampling error is usually set to ~5% [56] so our cut off point is erring on the more conservative. Some of the defined populations contain less than the desired 10% (typically 2–3 grains), and as these are not statistically significant within the wider distribution of grains in the sample they have been defined as outliers (not reflective of any of the populations) and to be more conservative that population is not used for D_e_ determination. For example, in samples BRY-6, CA-9 and JS-10 only, the upper dose population consisted of only 1–2 grains, which have been interpreted as residual dose outliers and the middle component has been used for D_e_ determination instead.

**Table 1:** OSL dating of quartz grains from mudwasp nests in Brremangurey, Lawley River and Lower Mitchell Falls areas, northwest Kimberley: dose rate data, equivalent doses, and age estimates.

Sample code	Motif, Stylistic Period	Portion of nest	Processed/ accepted grains	% Grains luminesce	Beta dose rate^a^ (Gy ka^-1^)	Sandstone (m)/panel/ gamma spec^b^	Field gamma dose rate^c^ (Gy ka^-1^)	Cosmic-ray dose rate^d^ (Gy ka^-1^)	Total dose rate^e^ (Gy ka^-1^)	Stimulation method^f^	Statistical Model^g^ (com/OD%)	Equivalent dose^h^^,^^I^ (Gy)	Age^j^ (ka)
***Lawley***													
**UP1A**	‘Cage-shape’ over Mambi Gwion anthropomorphic figures	Entire	660/170	28	0.944 ± 0.042	3/V/TOT-BG	0.234 ± 0.004	0.193	1.40 ± 0.05	SG-CW	FMM (2/50)	4.61 ± 0.12	3.28 ± 0.19
		Entire	300/22	7	0.944 ± 0.042	3/V/TOT-BG	0.234 ± 0.004	0.193	1.40 ± 0.05	SG-LMOSL	f:s	4.10 ± 0.16	2.92 ± 0.16
**UP1B**	Off-art	L1—outer	400/104	26	1.031 ± 0.043	3/V/TOT-BG	0.234 ± 0.004	0.193	1.49 ± 0.05	SG-CW	CAM	0.24 ± 0.01	0.16 ± 0.01
		L2—inner	200/66	33	1.031 ± 0.043	3/V/TOT-BG	0.234 ± 0.004	0.193	1.49 ± 0.05	SG-CW	CAM	0.44 ± 0.02	0.28 ± 0.02
		L3—inner	100/24	24	1.031 ± 0.043	3/V/TOT-BG	0.234 ± 0.004	0.193	1.49 ± 0.05	SG-CW	CAM	0.84 ± 0.09	0.60 ± 0.07
		L4—core stub	100/32	32	1.031 ± 0.043	3/V/TOT-BG	0.234 ± 0.004	0.193	1.49 ± 0.05	SG-CW	CAM	4.50 ± 0.66	3.09 ± 0.47
		L4—core stub	200/25	13	1.031 ± 0.043	3/V/TOT-BG	0.234 ± 0.004	0.193	1.49 ± 0.05	SG-LMOSL	f:s	4.67 ± 0.81	3.21 ± 0.14
***Brremangurey***													
**BRY-3**	Anthropomorphic figure, Wanjina Period	Entire	400/117	29	0.919 ± 0.041	3/H/ 2π	0.257 ± 0.004	0.200	1.41 ± 0.05	SG-CW	FMM (3/30)	0.99 ± 0.04	0.70 ± 0.04
**BRY-6**	Fish, Wanjina Period	Entire	600/56	9	0.916 ± 0.041	3/H/ 2π	0.240 ± 0.004	0.200	1.39 ± 0.05	SG-CW	FMM (3/40)	1.97 ± 0.32	1.42 ± 0.24
***The Caverns***													
**CA-7**	Anthropomorphic figure (Argula), Wanjina Period	Entire	700/95	16	1.040 ± 0.044	3.5/V/TOT-BG	0.257 ± 0.004	0.199	1.52 ± 0.05	SG-CW	FMM (3/20)	2.49 ± 0.13	1.63 ± 0.11
**CA-8**	‘Yam-like’ shape, Period unknown	Entire	700/150	21	0.782 ± 0.038	2.5/H/TOT-BG	0.241 ± 0.004	0.201	1.26 ± 0.05	SG-CW	FMM(3/50)	21 ± 1	16 ± 1
**CA-9**	Anthropomorphic figures, transitionary Gwion/Wararrajai Gwion Period	Entire	300/59	20	0.765 ± 0.038	2.7/V/TOT-BG	0.210 ± 0.004	0.201	1.21 ± 0.05	SG-CW	FMM (3/20)	0.63 ± 0.11	0.53 ± 0.08
**CA-MOD**	Off-art	Entire	900/178	20	1.014 ± 0.044	3.5/V/TOT-BG	0.257 ± 0.004	0.199	1.50 ± 0.05	SG-CW	CAM	0.23 ± 0.01	0.15 ± 0.01
		Entire	300/23	8	1.014 ± 0.044	3.5/V/TOT-BG	0.257 ± 0.004	0.199	1.50 ± 0.05	SG-LMOSL	f:s	0.19 ± 0.01	0.13 ± 0.01
***Upper Bush Spirit Camp***													
**JS-10**	Anthropomorphic figure, Wararrajai Gwion Period	Entire	400/126	32	0.965 ± 0.042	3/H/TOT-BG	0.222 ± 0.004	0.200	1.42 ± 0.05	SG-CW	FMM (3/30)	1.32 ± 0.04	0.93 ± 0.05
**JS-11**	‘Star-yam’, Period unknown	Entire	600/120	20	0.876 ± 0.040	3/H/2π	0.218 ± 0.004	0.200	1.33 ± 0.05	SG-CW	FMM (2/30)	0.85 ± 0.15	0.65 ± 0.12
***Lower Mitchell Falls***													
**LM-13**	Macropod, Wanjina Period	Entire	600/99	17	0.801 ± 0.039	3/H/ 2π	0.230 ± 0.004	0.200	1.26 ± 0.49	SG-CW	FMM (3/30)	6.44 ± 0.12	5.10 ± 0.24

^a^ Concentrations determined from beta counter measurements of dried and powdered sediment samples.

^b^ Thickness of sandstone rock directly above the nest in metres/Method for conducting the gamma spectrometry at the site—2π = nest that were sampled on the cave roof with no other gamma contribution within 1.5 m—assuming a 2π geometry TOT/BG using the Perspex screen to isolate the gamma contributions from areas other than the rock surface (see methods).

^c^ Determined from U, Th and K concentrations measured using a portable gamma-ray spectrometer at field water content.

^d^ Time-averaged cosmic-ray dose rates (for dry samples), each assigned an uncertainty of ± 10%.

^e^ Mean ± total (1σ) uncertainty, calculated as the quadratic sum of the random and systematic uncertainties. An internal dose rate of 0.03 Gy ka^-1^ is also included. All the nests measured were completely dry so no addition for water content has been added apart from the modern nest CA-MOD with a water content of 2 ± 0.2%.

^f^ The luminescence stimulation techniques applied to these samples SG-CW-OSL = single-grain continuous wave optically stimulated luminescence and SG-LM-OSL single-grain linearly-modulated optically-stimulated luminescence.

^g^ The statistical models used to determine the dose distribution between aliquots = f:s the ratio between the fast and slow components with values closest to unity used for De determination. FMM—Finite Mixture Model with 'com' = no. of components used and 'OD%' = overdispersion as a %, CAM = central age model.

^h^ Equivalent doses include a ± 2% systematic uncertainty associated with laboratory beta-source calibrations.

^i^ OSL signal measured using single-grains of quartz—with between 300–700 grains run per sample (depending on the size of the nest) with on average 22% of the grains emitting an acceptable luminescence signal.

^j^ Uncertainties at 68% confidence interval.

Using the portable gamma spectrometer, we calculated the gamma dose rate of the sandstone bedrock to be between 0.210–0.257 Gy/ka^-1^, with only small variations between the measurements for bedrock only verses bedrock plus rest of the cave. Using beta counting of the nest we estimated a beta contribution of between 1.07–1.41 Gy/ka^-1^, with a cosmic dose rate of between 0.193–0.210 Gy/ka^-1^, which provided a dose rate of 1.2–1.5 Gy/ka^-1^. The HRGS analysis of the two nests (UP1B and CA-MOD) provided dose rates of between 2.2–3.5 Gy/ka^-1^ for the ancient and modern nests respectively (Table 2).

**Table 2:** Total dose rate for the mudwasp nests based on high resolution gamma spectrometry (HRGS) data for UP1B and CA-MOD.

Sample code	Site	^238^U (Bq/kg)	^226^Ra (Bq/kg)	^210^Pb (Bq/kg)	^228^Ra (Bq/kg)	^228Th^ (Bq/kg)	^40^K (Bq/kg)	Cosmic dose rate^a^ (Gy ka^-1^)	Water content^b^ (%)	Total dose rate^c^ (Gy ka^-1^)
***The Caverns***										
**CA-MOD**	LR03D	52 ± 6	53 ± 8	120 ± 32	57 ± 20	43 ± 7	120 ± 106	0.193 ± 0.019	2 / 2 ± 0.2	3.5 ± 0.6
***Lawley***										
**UP1B**	UP01	54 ± 5	55 ± 6	54 ± 8	42 ± 4	43 ± 3	118 ± 31	0.199 ± 0.019	0 / 0 ± 0	2.3 ± 0.2

^a^ Time-averaged cosmic-ray dose rates (for dry samples), each assigned an uncertainty of ± 10%.

^b^ Field/time-averaged water contents, expressed as (mass of water/mass of dry sample) x 100. The latter values were used to calculate the total dose rates and OSL ages. All the nests measured were completely dry so no addition for water content has been added apart from the modern nest CA-MOD with a water content of 2 +- 0.2%.

^c^ An internal dose rate of 0.032 Gy ka^-1^ for 200 μm quartz is also included.

For these two mudwasp nests, we observed unsupported excesses in ^210^Pb and ^226^Ra (see Table 2). In the ^238^U chain of the modern nest there was a 125% excess in ^210^Pb compared to ^226^Ra and a smaller 2.7% excess in ^226^Ra compared to ^238^U. While in the ^232^Th chain we observed a 25% deficiency in ^228^Th compared to ^228^Ra. These measurements support the modern nature of the nest as the ^210^Pb excess is a recent effect from fallout in the last 100 years, the disequilibrium between ^230^Th and ^226^Ra has occurred within 600 years and any ^226^Ra excess still present suggests the sample is <6 kyr. As eight of the nests are older than 600 years the effects of disequilibrium between ^230^Th and ^226^Ra, and ^210^Pb excess would have decayed to negligible levels. However, only one nest is older than 6 kyrs so the remaining eight may have been affected by ^226^Ra excess. In contrast, the older UP1B sample has no ^210^Pb excess, negligible disequilibrium between ^238^U and ^226^Ra and only a very minor ^226^Ra excess of 1.8% suggesting that the sample is not modern but has not yet reached the 6 kyr half-life for the decay of ^226^Ra. Therefore, while the modern nest displays excesses, the older nest has only minor disequilibrium. The difference in total dose rate between the younger and older nests is ~1 Gy/ka due to the modern excesses. When the disequilibrium in the modern nest is modelled as a recent excess that has been adding steadily since time of deposition, a similar dose rate to the ancient nest is obtained. In addition, the ratio of ^210^Pb/^226^Ra provides a ^222^Rn emanation of just 2.3% since nest construction–this drops to 0.98% in the older nest. The high resolution gamma spectrometry technique applied to the nests provided a higher dose rate than the beta counting/*in situ* gamma method.

The initial bleaching test on a modern nest (CA-MOD), processed using the central age model (CAM), revealed that statistically there was no difference between the inner and outer grains from the nest (overdispersion = 9%) and that the luminescence signal was at a low level (0.23 ± 0.01 Gy equivalent to 150 ± 15 yrs). In addition, the age of the layers for the ancient fossilised nest (UP1B) increased with distance towards the core from Layer 1; at 0.24 Gy, Layer 2; 0.44 Gy, Layer 3; 0.84 Gy and finally the core; at 4.50 Gy, these D_e_ equate to ages of 160, 280, 600 years and 3.09 kyrs respectively, with the inner grains containing an average of 4.5 Gy more dose. Furthermore, there is agreement between the outer bleached layer in the ancient nest (0.24 ± 0.01 Gy) and the weighted mean of all the grains from the modern nests (0.23 ± 0.01 Gy). The distinct differences in dose between the layers of the nest suggest that the partial removal of the outer layer have not affected the signal retention of the grains within the core.

**Fig 7:**
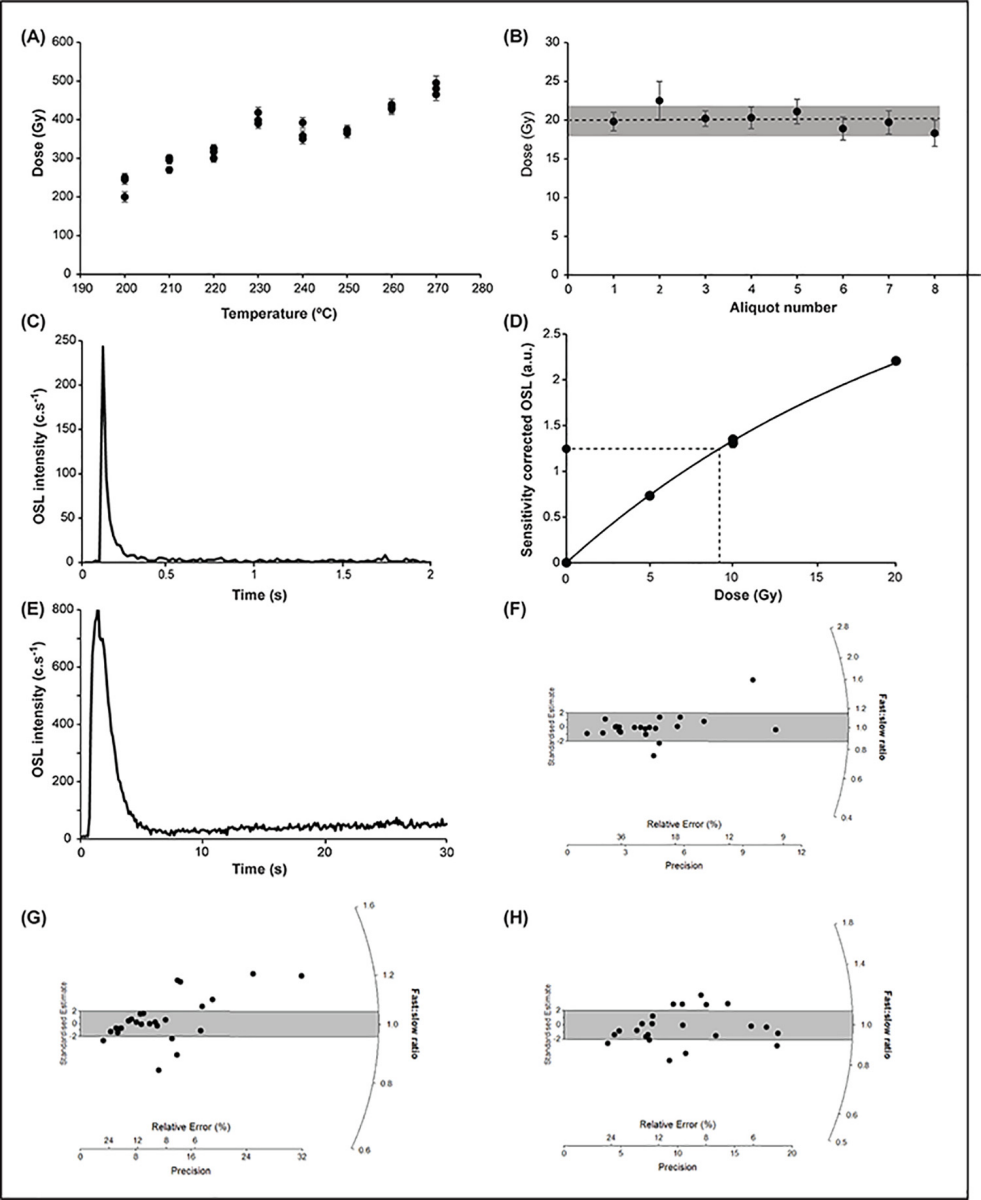
Luminescence characteristics of the quartz grains found in the mudwasp nests. (A) a preheat plateau test for sample UP1A. Fresh aliquots of 180–212 μm quartz were run using different preheat temperatures from 200–270°C and demonstrate a slight plateau at ~250°C, this was chosen as the preheat temperature for the samples. (B) A dose recovery test using 8 aliquots of fresh quartz and the chosen preheat temperature of 250°C. All aliquots recovered the 20 Gy surrogate dose (dashed line) within 2 σ error (shaded box). (C) SG-OSL shine down for a continuous wave laser stimulation of 2 secs. (D) The resulting dose response curve from the same grain as shown in (C) with a D_e_ of 9.2 Gy. (E) LM-OSL shine down over a stimulation period of 30 s. Note the dominance of the fast component (first 5 s) and the smaller slow component (last 5 s). (F-G-H) LM-OSL D_e_ results for sample F- UP1B, G—UP1A and H—CA-MOD plotted as a ratio of fast:slow components (rather than their D_e_ values) on the radial plot and centred on unity. Therefore grains with a similar fast and slow component D_e_ values will be centred in the middle of the plot. The 2 σ range depicted by the grey shading indicates the grains closest to unity and their corresponding D_e_ values were used to obtain an LM-OSL age.

The LM-OSL results for samples UP1A, UP1B and CA-MOD for both the fast and slow components provided a ratio of f:s signals for these nests with 57–86% of grains being close to unity (Fig 7F–7H). However, only a small number of grains had a usable fast and slow component signal with low percentages of accepted grains (5–6%). While a useful technique, the low acceptance makes this approach unfeasible for the smaller nests with minimal amounts of quartz. Grains containing the highest doses and exhibiting a f:s ratio close to unity provided D_e_ values that were close to the estimate using FMM on the CW data.

The results for the AMS ^14^C dating of beeswax found over art and organic material found within a mudwasp nest are presented in Table 3. OSL and AMS ^14^C samples were obtained from the same two motifs; one at LR03D (1630 ± 110 yrs and 687–884 yr cal. BP) and one at Brremangurey (OTB01) (700 ± 40 yrs and 346–341 yr cal BP, respectively). While the results only provide minimum age estimates for the art (and therefore do not need to agree), they are coeval within error margins. These comparisons were only conducted on younger art styles as samples from the older art styles were not available.

**Table 3:** Radiocarbon ages and supporting data for the beeswax and mudwasp nest (after [57,58])^a^.

Sample code	Motif, Stylistic Period	S-ANU#/ ANSTO Code	Other ID	δ^13^C per mil	Percent Modern Carbon pMC	D14C	^14^C age	Age yr cal BP^b^^,^^c^
LR03S-01	Anthropomorphic figure (Argula), Wanjina Period	33109	9551	-23 ± 2	85.83 ± 0.34	-141.7 ± 3.4	1230 ± 35	687–884
LROIC-2	Anthropomorphic figure, Wararrajai Gwion Period	33110	9978	-13 ± 2.0	85.20 ± 0.30	-148.0 ± 3.0	1285 ± 30	666–771
LROIC-3	Anthropomorphic figure, Wararrajai Gwion Period	33111	9979	-14 ± 2.0	85.17 ± 0.30	-148.3 ± 3.0	1290 ± 30	664–770
BRY-3	Anthropomorphic figure, Wanjina Period	OZQ990	-	-27.3 ± 0.1	98.06 ± 0.32	-	155 ± 30	346–321

^a^ δ^13^C values are the AMS machine quoted values and are used to correct the age. They can differ from IRMS results.

The quoted age is in radiocarbon years using the Libby half-life of 5568 years and following the conventions of Stuiver and Polach [59].

Radiocarbon concentration is given as percent Modern Carbon and conventional radiocarbon age.

Sample preparation backgrounds have been subtracted, based on measurements of samples of ^14^C-free CO_2_.

^b^ Calibrated using the CALIB 7.0.4 program [60].

^c^ Calibrated using IntCal 13 curve from OzCal 4.2 [61].

The oldest age estimate was obtained for the fossilised mudwasp nest overlying an elongated ‘yam-like’ motif with a bifurcated ‘root’ or ‘tail’ (CA-8, Fig 5D, Table 1), dated to a minimum 16 ± 1 ka. Minimum age estimates of 3.3 ± 0.2 were obtained indirectly for two Mambi Gwions (UP1A, Fig 5B, Table 2) and 0.53 ± 0.08 ka (CA-9, Fig 5C, Table 1), for an anthropomorphic figure from a transitionary Gwion/Wararrajai Gwion Period. The anthropomorphic figure with a distinctive triangular ‘robe’ shape returned a minimum age estimate of 0.9 ± 0.1 ka (JS-10, Fig 5A, Table 1), which also provides the only maximum age for a motif, an orange echidna (Fig 5A). The large red anthropomorphic figure with the ‘shocked’ headdress returned a minimum age estimate of 0.7 ± 0.1 ka (BRY-3, Fig 4C, Table 1) while the AMS ^14^C date for the whole nest over this figure was 346–321 cal BP years (BRY-3, Table 1). AMS ^14^C minimum age estimates of 666–771 yr cal. BP (LRO3C-2, Fig 5E, Table 1) and 664–770 yr cal.BP (LRO3C-3, Fig 5E, Table 3) were also obtained from beeswax resin overlying a Wararrajai Gwion figure. A minimum age estimate of 650 ± 10 years was returned for the ‘star-yam’ motif (JS-11, Fig 4A, Table 1). Two different dating techniques provided minimum age estimates for the *Argula*: 1.6 ± 0.1 ka (CA-7, Fig 4B, Table 1) using OSL for an overlying mudwasp nest and 687–884 yr cal. BP (LR03S-01, Fig 4B, Table 3 [S-ANU 33109.9551]) for AMS ^14^C on wax resin. The two zoomorphic figures sampled, returned a minimum age estimate of 1.4 ± 0.5 ka (BRY-6, Fig 4D, Table 1) for the bi-chrome fish, while the large poly-chrome macropod provided a minimum age estimate of 5.1 ± 0.2 ka (LM-13, Fig 4E, Table 1).

## Discussion

The dose rate for the nest only using the HRGS method had a large gamma dose rate (1.051–1.678 Gy/ka^-1^) that far exceeds that estimated by the *in situ* method (~0.23 Gy/ka^-1^) and that estimated by other authors for a similar geology (e.g., [37] ~0.31 Gy/ka^-1^, [57] ~0.4 Gy/ka^-1^). We attribute this discrepancy to the difference in material used–the HRGS was applied to the nest only while the dosimetry of the *in situ* method (portable gamma spectrometry and beta counting) was damped down by the inclusion of lower-dose sandstone. The beta dose rate according to the HRGS method is slightly higher than the beta counting result. We attribute this to the use of a small amount of parent material in the beta counting sample to provide a more accurate representation of the beta contribution. Thus, if the gamma measurement derived from the HRGS is reduced to that determined by the *in situ* method, the total dose rate for both techniques are in agreement (1.5 and 1.4 Gy ka^-1^ respectively). This total dose rate is lower than estimated by Yoshida *et al*. [37] despite having similar estimations of the gamma dose rates on the sandstone bedrock. This difference is derived from the beta contribution in the nests (1.4–1.7 verses ~2.60 Gy/ka^-1^, respectively), which we attribute to a different sedimentary source for the mudwasp nest from different drainage basins (Lawley and Mitchell River verses the King Edward River). The estimate for radon emanation according to the HRGS result suggests that radon loss on the surface of the sandstone is not contributing to the nest disequilibrium and therefore no correction has been made to account for this loss. However, ^226^Ra excess may have been an issue for the eight samples that are <6000 ka. To account for this disequilibrium and the limitations of high resolution gamma spectrometry for determining the dosimetry based solely on gamma emitting radionuclides, a 2% error margin was added to the dose rate for these samples to cover the range of difference with and without this excess.

The bleaching tests conducted on the modern off-art nest (Fig 3E and 3F), indicate that most of the grains in the nest are bleached prior to nest construction and this is confirmed by the high fast:slow ratios from the LM-OSL data and the number of grains close to unity (Fig 6F–6H). The fast component is known to bleach within a few seconds [38] but the slow component can take a few hours to be completely removed [62]. Therefore, if both the fast and slow component retain a similar dose, it implies that the grains must have received at least a few hours bleaching prior to burial. This bleaching occurred either during exposure on the sediment surface prior to sampling, during sampling, flying and nest construction by the mudwasp, or during exposure on the outside of the nest. Therefore, most grains contain negligible residual OSL signal prior to their burial so the signal measured in the laboratory represents the dose accumulated since nest construction. The agreement between the D_e_ values for the modern nest and the outer layer of UP1B support this prior bleaching assumption and provide a baseline of bleaching for grains found on the outer layers of the nests. This baseline of ~0.2 Gy agrees with the lower component of the FMM for all the nests ranging from 0.15–0.34 Gy. Furthermore, the agreement between the D_e_ derived from the LM-OSL f:s ratios close to unity for sample UP1A and the D_e_ derived from the upper component of the FMM using CW provide confidence for the use of CW FMM for the smaller nests.

The measurements on the individual layers of the ancient nest (Fig 3A–3D) indicate that a shielded inner core exists within the nest, which has accumulated more dose than any of the other layers tested, and that the amount of OSL signal decreases with distance towards the surface of the nest. This inner core appears to be unaffected by the absence of the outer coating, which provides confidence for the signal retention of nests with a partial absence of this layer (e.g. CA-9, JS-10 and JS-11). Thus, a surprisingly small thickness of nest material was required before the grains are effectively ‘buried’. Based on these findings, the use of small nests is valid. The number of fast:slow LM-OSL ratios close to unity for the core of the ancient nest (Fig 7F) indicates that the signals in these grains were not derived from a residual OSL signal but rather have accumulated during a shielded or burial period. This conclusion is supported by the higher number of unity grains in the core compared to the other two nests from all layers. The latter grains with a larger number of lower f:s ratios indicate that they have intermittently experienced brief bleaching episodes that have removed some of the fast but not the slow component signal. We interpret these grains as being located in the outer layers of the nest.

The isolation of the core grains using a FMM was successful in all nine samples with 2–3 dose populations identified. For the majority of the samples we interpreted the lower dose population in each sample as the ‘bleached’ outer layers of the nest, and the middle dose population as the grains that received some bleaching, while it was the outer layer and some signal accumulation during its part-burial. Therefore, the upper population of grains were considered to be the ‘unbleached’ inner core of the nest, and the population that best represents the signal accumulated during the burial period and a minimum age for the underlying art. For eight of the nests processed, the use of over 100 grains is a statistically significant number for D_e_ determination. For the remaining nest, 56 grains were accepted. The agreement between the D_e_ of the grains with a high doses and f:s ratios close to unity and the FMM upper component indicates that most of these grains previously underwent substantial bleaching prior to burial.

Samples BRY-3, BRY-6, JS-10 and CA-7 are all identified as containing an upper dose population of between 2–4 grains that have been interpreted as outliers. Due to the bleaching tests conducted on the modern nest (CA-MOD) these grains cannot be attributed to partial bleaching. Nor can they be attributed to contamination from the sandstone bedrock as quartz grains with a low sensitivity from a lack of bleaching or dosing cycles that changed dramatically during the measurement process were not observed in SG-OSL processing. Instead, we attribute these outliers as contaminating grains from inside the ochre paint or from the surface of the rockshelter prior to nest construction. As these outliers are not observed in all the samples and seem to predominate in the Brremangurey rockshelter, we speculate that they might be aeolian in origin, having blown into the rockshelter and adhered to the shelter walls. The limited number of these grains precludes them from being a significant problem.

It can be observed that the upper dose population contains the widest range of precision between the D_e_ values and the most precise grains. This is due to the presence of very bright and very dim grains, providing high and low precision, respectively. This is a typical observation in a radial plot at the same D_e_ value, with the lower precision dim grains still providing a similar D_e_ value but due to the reduction in counting statistics produced by the low counts is the signal harder to measure and therefore less precise. The presence of dim grains in the upper dose values is not a reflection of inaccurate data but rather a typical characteristic of luminescence dose populations.

The data from the off-art sample, UP1B, provides very useful cautionary evidence for the use of sections of nest that extend off the art. This section of the nest appears older than the on-art sample—UP1A. Rather than the nest being constructed before the art, it would seem that this section of the nest has been constructed on an older stump and therefore provides an older age using this technique. The UP1A section of the nest was probably constructed at a similar time, but as this section was not constructed on the older stump it provides a more reliable minimum age for the art. Therefore, we caution practitioners against using any section of the nest that is not directly on the art for fear of incorporating older off-art stumps in to the D_e_ determination.

Using this technique to determine D_e_ values and age estimates, the OSL results agree with independent age estimates obtained from the AMS ^14^C technique (Tables 1 and 3) in specific cases. While these comparisons could only be conducted on younger art styles, the agreement provides a measure of confidence in relation to the upper age range results where lack of suitable samples meant that comparisons could not be made.

As we were unable to sample under the art, the mudwasp nests sampled were all on top of the art (apart from a single example where a motif partially overlay the sampled nest UBSC01), the resulting age estimates represent minimum ages. These estimates are useful for formulating a baseline for the relative stylistic sequence, but they do not provide us with an upper age range or direct dates for individual motifs. The use of the entire mudwasp nests is certainly not ideal but this became necessary when larger nests were unavailable. However, with the absence of larger nests on top of art, smaller nests can provide meaningful data if a conservative approach is adopted.

### Implication of the results for the chronology of rock art in the northwest Kimberley

The minimum age estimate of 16 ± 1 ka for an elongated ‘yam-like’ motif with a bifurcated ‘root’ or ‘tail’ (Fig 5D) makes it one of the oldest *in situ* rock art motif thus far established for any Australian rock art assemblage. However, the style of the motif differs from the Gwion Period figure claimed to be of similar age. While the large size and colour used by the artist to produce the motif are all typical of those used on IIAP motifs, the form and subject matter are not, nor is the infill as roughly applied as some IIAP motifs making it difficult to assign the motif to one of Walsh’s stylistic classifications. Two similar motif forms were recorded at other locations in the northwest Kimberley but neither is painted in a manner that assisted with the classification of the sampled motif. The rarity of the motif type makes it impossible to extrapolate from this result to identify stylistically similar motifs of the same age across the Kimberley assemblage.

On their own, little can be gleaned from the minimum age estimates of 3.3 ± 0.2 ka for the ‘cage-shape’ motif (Fig 5B) (and indirectly, the underlying Mambi Gwion figures), and 0.53 ± 0.08 ka for the fossilised nest built over the headdress of a transitional Wararrajai Gwion Period figure (Fig 5C). Our analysis of the superimposition of rock art styles undertaken during the archaeological project supports Walsh’s conclusion that Mambi Gwion depictions predate Wararrajai Gwion figures although it is possible that the styles were contemporaneous in rare instances. It is more likely that the results reflect the differences in the timing of the nest construction rather than the emergence of each style. However, the minimum age estimate for the figure in the frieze (Fig 5C) is stylistically similar to the figures depicted as crew members in paintings of two canoes documented in a site adjacent to the Mitchell River [11]. If in fact, the canoe represents outsiders visiting the Kimberley region in the past, the minimum age estimate of 0.53 ± 0.08 ka would also provide a minimum timeframe for maritime contact and much earlier than Macassans are known to have visited the coast.

A more recent minimum age estimate of 0.9 ± 0.1 ka for the large anthropomorphic figure with a distinctive triangular ‘robe’ shape (Fig 5A) with features most commonly found in the Wararrajai Gwion Period, such as straight limbs and body depicted in full frontal position, adds little to clarify the relationship between the various Gwion styles. This nest also provides the only maximum age estimate obtained during the project. A section of an echidna motif attributed to the Painted Hand Period (Fig 6) clearly overlies part of the processed nest (although the section of the nest immediately underlying the motif was left in place). Therefore, the echidna motif has to have been painted after 0.9 ± 0.1 ka. This is a much more recent date than has previously been suggested for this style. The Painted Hand Period is usually considered to predate the Wanjina Period [14]. These results demonstrate that at least some styles amongst the more recent art were contemporaneous.

The simplicity of the form and the splayed foot position of the large, red anthropomorphic motif (Fig 4C) at Brremangurey are typical of the Wanjina Period. This classification is supported by the relatively recent OSL (0.78 ± 0.1 ka) and AMS ^14^C (346–321 cal BP) age estimates. The slight disparity between the two dates produced for the same nest can be explained by the difference in the actual material dated: the OSL age estimate was obtained from grains in the layer of nest closest to the substrate while the AMS ^14^C age estimate was obtained using only the fine grained material from the whole nest where there is more chance of modern carbon being included [23].

Similarly, the nest overlying the typical Wanjina Period, bi-chrome fish (Fig 4D) painted at Brremangurey returned a minimum age estimate of 1.6 ± 0.6 ka, in the expected range of ages for this style. The sample also provides a minimum age estimate for the two underlying polychrome anthropomorphic figures typical of the Painted Hand Period, but again, there is no way to estimate the difference in the timing of the two painting episodes.

**Fig 8:**
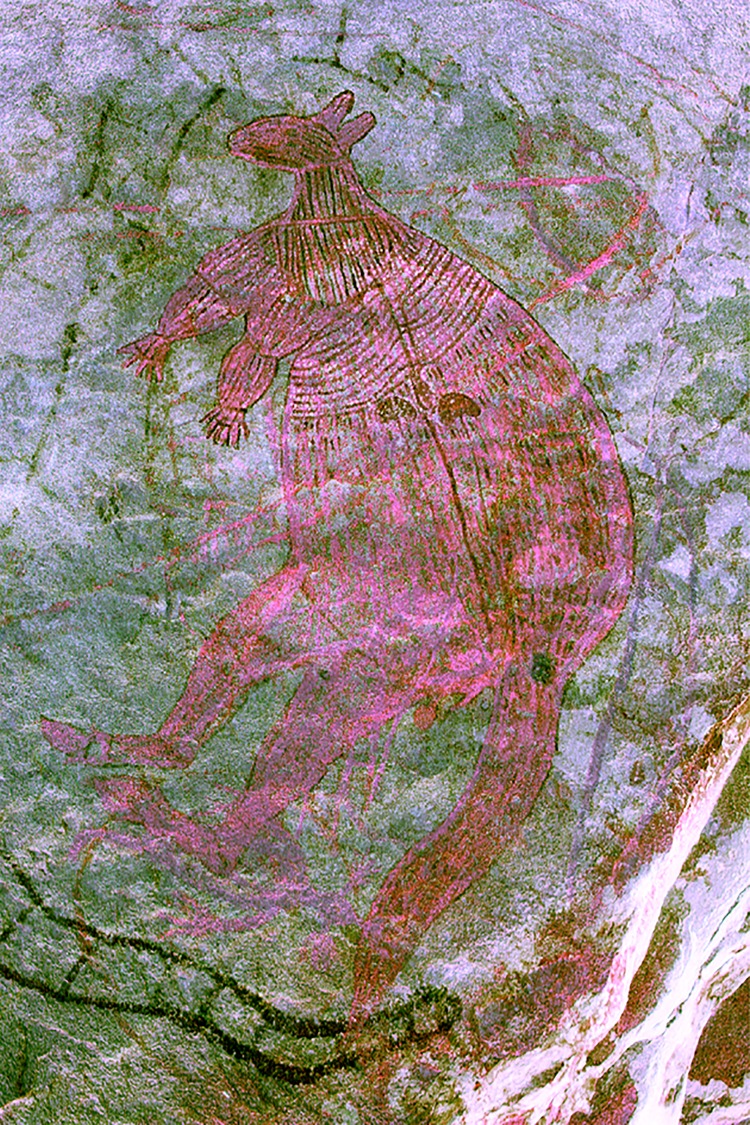
Wanjina Period macropod (LM-13) depicted with spine and kidneys, photograph digitally enhanced using D-Stretch.

The oldest date for a Wanjina Period motif prior to our project was a AMS ^14^C date of 3,780 ± 60 yr cal. BP [9] for a Wanjina head made from beeswax pellets, so the minimum OSL age estimate of 5.1 ± 0.2 ka for a nest overlying a Wanjina Period macropod (Fig 4E and Fig 8) was unexpected and pushes back the timing of the emergence of the Wanjina style by more than 1000 years. The body and limbs of the macropod motif sampled are divided into sections in a manner typical of the Wanjina Period with the entire motif infilled with fine longitudinal lines, while white dotting has been added to the shoulders, forearms and kidneys.

The depiction of a backbone and kidneys is similar to the manner used in the X-ray art of the Arnhem Land region to the east. Motifs with simple X-ray elements have been recorded in three Gwion Period figures during the project, but are more common in the Painted Hand Period motifs where animals are sometimes depicted with backbone and ribs. Similarities between the early rock art assemblages of both regions have been flagged, but it may well be that interactions between the two regions have extended into the more recent past. A much larger Kimberley rock art sample would need to be analysed before such a claim could be supported.

The correlation between the emergence of the Wanjina Period style and the ‘Early X-ray’ style paintings in Arnhem Land at around 6 kya [60] adds weight to hypothesized connections between the two regions [63]. ‘Early X-ray’ art is an ancestral form of the ‘Complete Figure Complex’, which emerged around 4 kya [64–65]. This age range is supported by two radiocarbon ages of 4040 ± 80 BP [66] and 4460 ± 80 BP [67] for a beeswax turtle image from Gunbilngmurrung in Arnhem Land.

Finally, the difference in age estimates for the *Argula* figure (Fig 4B) with a minimum OSL age estimate of 1.6 ± 0.1 ka and an AMS ^14^C age estimate of 687–884 yr cal. BP can again be explained by the difference in the material dated. The nest and the beeswax resin appear to have been laid down at slightly different times but provide a minimum age range for production of this type of spirit figures, which sits comfortably within the range of direct AMS ^14^C dates (433–656 cal. CE, 691–989 cal. CE and 679–1148 cal. CE) obtained by Morwood *et al*. [9] for stylistically similar beeswax or charcoal *Argulas*. The dating of two different samples using two different techniques highlights the problems inherent in attempting to develop a temporal framework based on a small sample of minimum age estimates.

The challenge of classifying some of the motifs covered by the sampled nests highlights the problem we encountered more broadly during the project: a large proportion of the motifs we recorded do not fit neatly into one or another classificatory style. Many motifs had stylistic elements common to more than one stylistic classification suggesting the gradual introduction of new styles and the concomitant demise of others, indicating that more fine-grained analysis will need to be done to refine various Kimberley stylistic sequences.

The difficulties inherent in the use of style as a chronological marker have long been flagged [68–69] and are illustrated by the recent date obtained for a Painted Hand Period motif. Lorblanchet and Bahn [70] have argued that, following the introduction of new dating methods such as AMS ^14^C, rock art studies would enter a ‘post stylistic era’. Such an extreme approach however, would mean that each and every motif would have to be dated if it was to be incorporated into a temporal framework. The approach we have adopted using stylistic classifications complemented by dating techniques is likely to provide the most useful framework with the latter used to ‘probe and anchor stylistic sequences’ and the former to ‘identify problems or inconsistencies in … dating’ [71].

The single, older minimum age estimate we were able to obtain during the project, despite extensive surveys, highlights the limited availability of ancient mudwasp nests suitable for OSL sampling. In many cases, taphonomic processes have led to the gradual breakdown of nests leaving only nest stubs comprising limited unbleached grains. The single older minimum age estimate we obtained for the ‘yam-like’ motif provides supporting evidence for the claim that rock art was produced in the Kimberley during the terminal Pleistocene [20]. Further, the oldest of the minimum age estimates (16 ± 1 ka) sits well inside the temporal framework proposed for the emergence of Arnhem Land rock art between 26,913–28,348 years, calibrated BP [24].

## Conclusion

**Fig 9:**
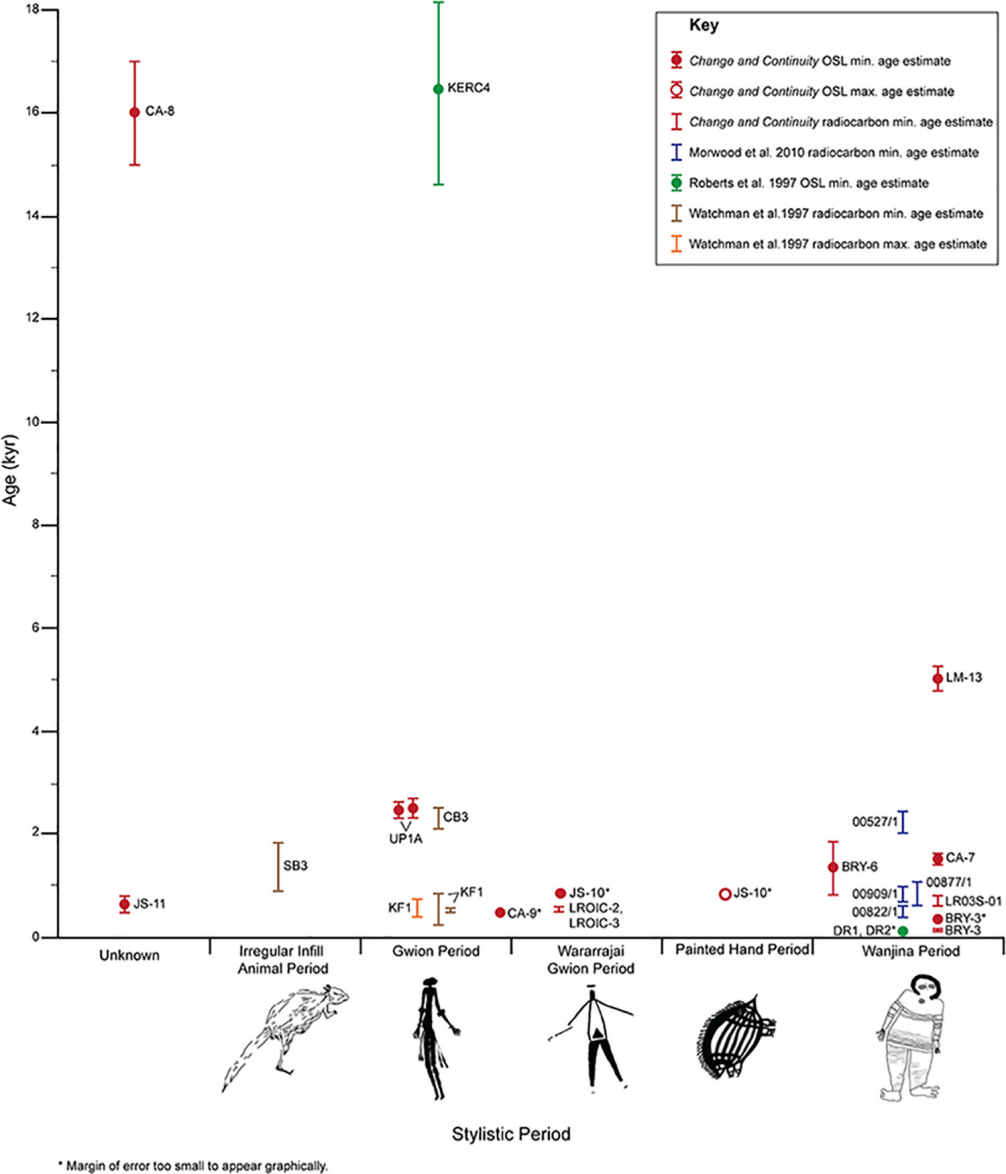
Summary of 26 results from Kimberley rock art dating projects.

The results we have obtained demonstrate the limitations of relying on minimum age estimates alone to establish a temporal framework for Kimberley rock art. Many more samples will have to be processed before a sound chronology can be proposed. Nevertheless, our results provide support for earlier claims of a Pleistocene origin for the production of rock art in the region. However, the four rock art dating projects undertaken in the Kimberley thus far (Fig 9) have returned only two Pleistocene OSL minimum age estimates. While these results could be interpreted as signifying relatively recent origin for the art assemblage in the Kimberley, alternatively, they may well be a reflection of the paucity of ancient mudwasp nests, mineral skins or resin available to sample.

Any dating program aims to identify and sample classic exemplars of accepted stylistic phases so that results can be extrapolated to similar motifs across the broader assemblage. Although few classic motifs were found to be suitable for sampling during this project, extrapolation from the results of one sampled figure to a group of stylistically similar figures has provided a means to propose a minimum date for the timing of potential maritime incursions into the region.

Our results have pushed back the timing of the emergence of the Wanjina Period and opened up the possibility of contact between past inhabitants of Arnhem Land and the Kimberley as recently as the mid-Holocene. Moreover, the results have identified at least one case of the co-occurrence of recent Kimberley rock art stylistic phases previously considered sequential, thus signalling that the relationships between styles may not necessarily be temporally separated. The potential for different contemporaneous art styles performing separate functions then becomes a possibility emphasising the need for ongoing fine-grained rock art analyses and contextual studies. While the results are by no means conclusive or extensive, they provide a significant step towards our understanding of the complexities of Kimberley rock art.

## Supporting Information

S1 Fig LR03D floorplan with location of samples CA-7, CA-8, CA-9, LR03S-01, LROIC-2 and LROIC-3. (TIF)

S2 Fig Brremangurey (OTB01) floorplan with location of samples BRY-3 and BRY-6. (TIF)

S3 Fig LMR02C floorplan with location of samples LM-13. (TIF)
