# Supplementary figures and images for: Into the Past: A Step Towards a Robust Kimberley Rock Art Chronology
Source: PLoS One. 2016 Aug 31;11(8):e0161726. doi: 10.1371/journal.pone.0161726 (PMC5006964; doi:10.1371/journal.pone.0161726)

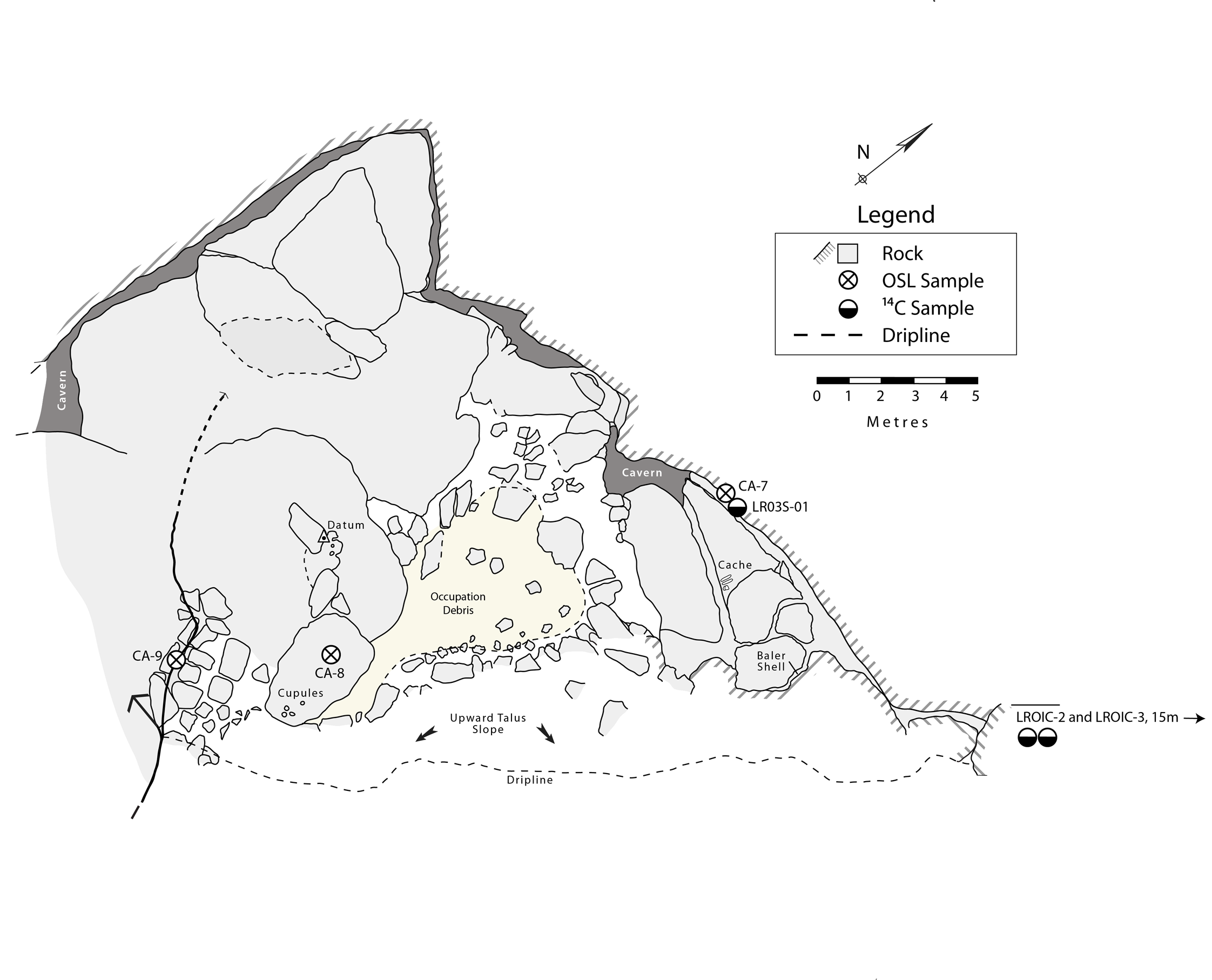

Supplement: S1 Fig — (TIF) [file pone.0161726.s001.tif]

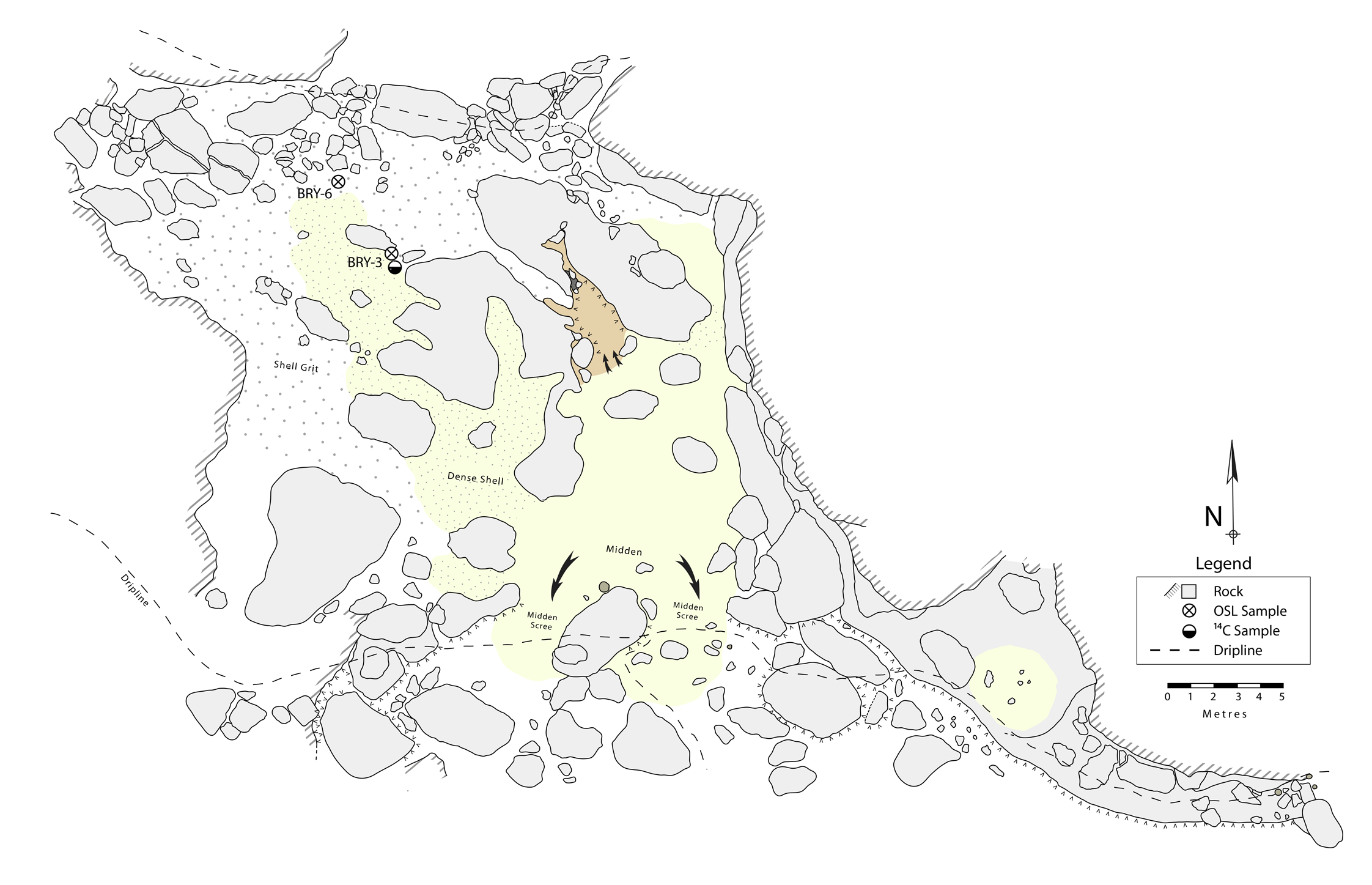

Supplement: S2 Fig — (TIF) [file pone.0161726.s002.tif]

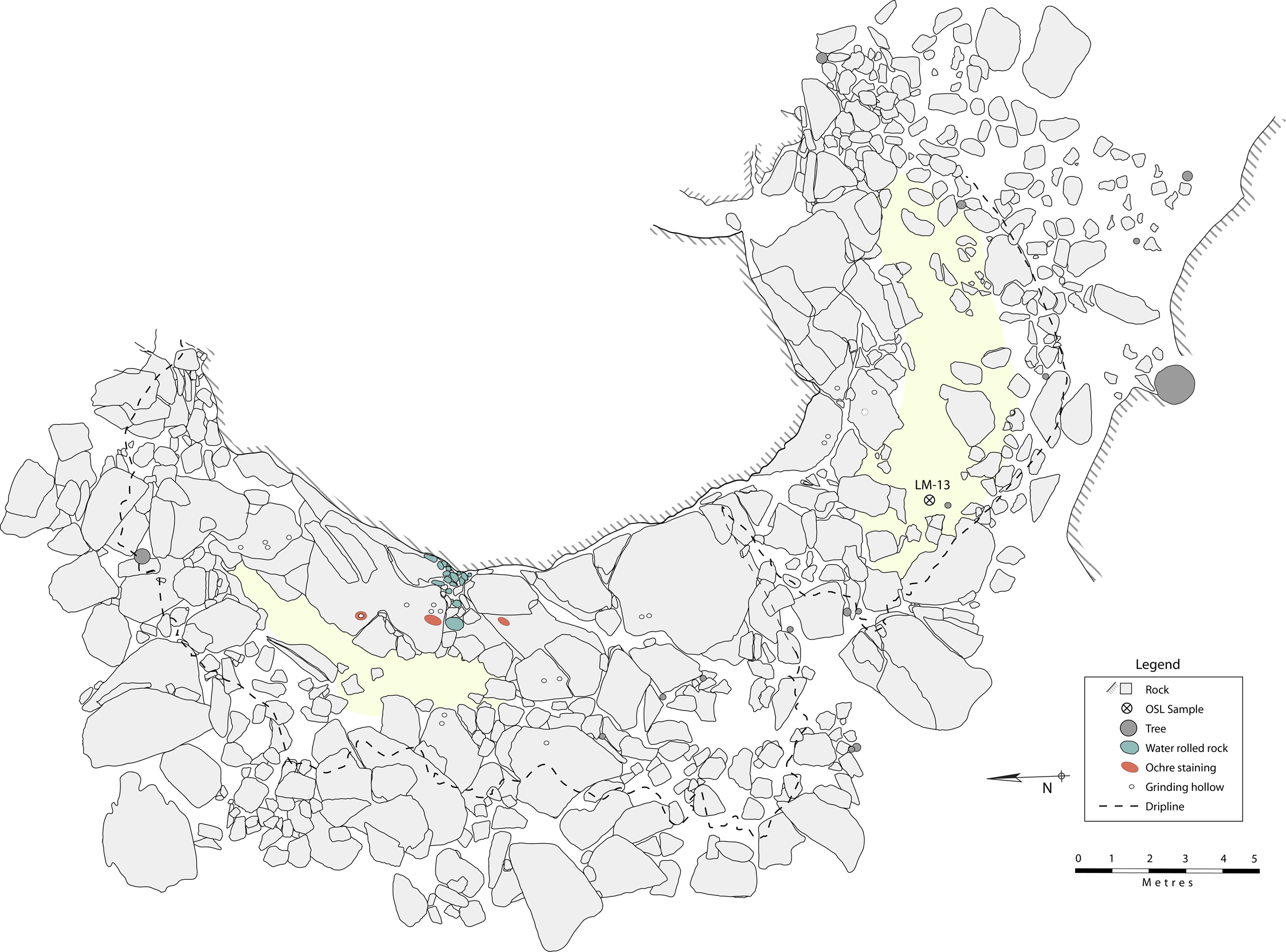

Supplement: S3 Fig — (TIF) [file pone.0161726.s003.tif]
